# Pangenome of *Streptomyces sampsonii* and Relatives Highlights Horizontal Gene Transfer and Secondary Metabolism in Environmental Adaptation and Ecological Significance

**DOI:** 10.1002/ece3.74350

**Published:** 2026-09-15

**Authors:** Boyang Zhang, Shuo Ji

**Affiliations:** ^1^ School of Architecture and Art Hebei University of Architecture Zhangjiakou Hebei China; ^2^ Department of Energy Engineering Hebei University of Architecture Zhangjiakou Hebei China

**Keywords:** BGCs, comparison genomic, HGT, pangenome, *Streptomyces sampsonii*

## Abstract

*Streptomyces sampsonii* is a promising biocontrol bacterium, but its genomic basis of adaptation and secondary metabolism remains unclear. Here, we present a chromosome‐level genome assembly of *S. sampsonii* (7.20 Mb, 6015 protein‐coding genes) and perform comparative analyses with 95 related *Streptomyces* species. Phylogenomic and synteny analyses revealed its closest relationship with *S. albidoflavus* , while extensive structural variations distinguished more distant lineages. Pangenome analysis uncovered 84,178 gene clusters, with pan_shell and pan_cloud genes predominantly enriched in xenobiotic biodegradation, metabolism, and antibiotic biosynthesis, highlighting their roles in ecological adaptation and biocontrol potential. Biosynthetic gene cluster (BGC) analysis identified numerous NRPS, PKS, and terpene pathways, many of which belong to pan_shell and pan_cloud regions, suggesting dynamic evolutionary origins. We further detected 66,260 horizontally transferred (HGT) genes, including 438 in BGCs, underscoring HGT as a major driver of metabolic innovation. Together, these findings provide novel insights into the genomic diversity, adaptive capacity, and secondary metabolic potential of *S. sampsonii* and its close relatives.

## Graphical abstract

This study presents a chromosome‐level genome assembly of *Streptomyces sampsonii* and a pangenome analysis of 95 strains, revealing a highly expansive “open” pangenome. Our results highlight that horizontal gene transfer (HGT) and accessory gene variations drive the evolution of secondary metabolism and environmental adaptation. These findings provide key insights into the genomic plasticity and ecological success of the *Streptomyces* genus.

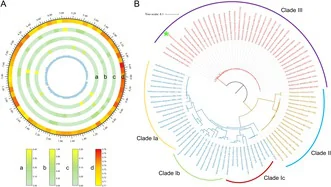

## 1 Introduction

*Streptomyces sampsonii* belongs to the family Streptomycetaceae and the genus *Streptomyces* (Stackebrandt et al. 1997). It is a strictly aerobic, Gram‐positive bacterium characterized by highly branched substrate mycelia (Rosenberg et al. 2014), and is commonly found in soil (Hodgson 2000). *S. sampsonii* exhibits antagonistic activity against fungal pathogens, including the genera *Alternaria* Nees and *Phomopsis* Sacc. & Roum (Malviya et al. 2009), as well as the species *Candida albicans* (C.P. Robin) Berkhout, *Aspergillus niger* van Tieghem, *Microsporum gypseum* (E. Bodin) Guiart & Grigoraki, *Trichophyton* sp. (Castell.) Sabour. (Jain and Jain 2007), and *Rhizoctonia violacea* (Tul.) Pat. (Li et al. 2014). Previous studies have shown that bioactive compounds produced by *S. sampsonii* have important applications in various fields. The strain *S. sampsonii* KK1024 produces insecticidal compounds capable of controlling root‐knot nematodes (Kim et al. 2011). In addition, crude extracts of metabolites from *S. sampsonii* LGMB262 have been reported to inhibit the growth of glioblastoma multiforme (GBM) (Savi et al. 2015). These findings highlight the potential of *S. sampsonii* as an important microbial resource.

Currently, the control of crop diseases mainly relies on chemical measures. Although chemical pesticides are effective in protecting plants against pests and pathogens, their side effects on humans and animals, as well as their impact on environmental pollution, cannot be ignored. The use of microorganisms for disease control can effectively reduce the negative impacts caused by traditional pesticides in forestry and agriculture. Given the important role of *S. sampsonii* in plant responses to biotic stress, elucidating its genomic information will facilitate a deeper understanding of its metabolic potential and application value in biological control. The genome represents the most comprehensive repository of genetic information for a species, providing the foundation for exploring its potential in secondary metabolite biosynthesis, environmental adaptation, and evolutionary history. To date, many genomes within the genus *Streptomyces* have been assembled. For example, *Streptomyces coelicolor* A3(2) was the first actinomycete to have its genome completely sequenced, revealing numerous genes involved in responses to environmental stimuli and stress (Bentley et al. 2002). The genome assembly of *Streptomyces ambofaciens* indicated that DNA rearrangements primarily affect the terminal regions of its linear chromosomes (Annabelle and Pierre 2016). Similarly, Omura et al. assembled the genome of *Streptomyces avermitilis* and predicted its capacity for secondary metabolite production (Ōmura et al. 2001). However, genomic studies of *S. sampsonii* remain limited, and the genetic mechanisms underlying its secondary metabolite biosynthesis, diversity formation, and ecological adaptation have not yet been systematically elucidated. Therefore, it is necessary to conduct more comprehensive genome assembly and comparative genomic analyses of this species.

A single genome can only reflect the genetic information of a representative individual, while extensive gene gains, losses, and structural variations commonly exist among different individuals or populations, which are often overlooked. The concept of the pangenome, representing the entire gene repertoire of a species or population, was first proposed in bacteria (Tettelin et al. 2005). Analysis of pangenomes enables a more comprehensive understanding of genetic diversity and evolutionary dynamics within species. For example, the pangenome analysis of *Vibrio cholerae* revealed the evolutionary trajectory of the pathogen and its geographical movements (Domman et al. 2017). Studies of bacterial and archaeal pangenomes have also uncovered novel anti‐phage defense systems (Doron et al. 2018). In a pangenome study of 17 pathogenic *Leptospira interrogans* strains, which cause the zoonotic disease leptospirosis, subcellular localization of proteins derived from the complete genome set was used to predict their surface‐exposed proteins (PSEs), providing valuable candidates for vaccine development (Zeng et al. 2017). Pangenome approaches have gradually become an important tool in bacterial comparative genomics, not only helping to better explain the genetic basis of trait differences among species, but also enabling the discovery of functional genes associated with environmental adaptation, pathogenicity, and metabolic potential. These insights, in turn, provide critical clues for understanding the ecological roles and applied value of different species.

Horizontally transferred genes refer to exogenous genes acquired through horizontal gene transfer (HGT) events, which overcome phylogenetic constraints and enable the exchange of genetic information across different species or populations. Compared with vertical inheritance, horizontal transfer provides bacteria with a rapid means of acquiring new functions, allowing them to quickly gain antibiotic resistance, metabolize novel substrates, or adapt to extreme environments (Arnold et al. 2022). At present, studies on horizontally transferred acquired genes (HGT‐acquired genes) in bacteria are well established, and abundant evidence has demonstrated that HGT plays a central role in bacterial evolution. It not only drives the formation of metabolic diversity but also facilitates the dissemination of virulence factors and antibiotic resistance genes. For instance, the exchange of virulence and resistance genes through HGT in *Vibrio harveyi* enhances its pathogenicity and resistance (Deng et al. 2019). Similarly, HGT in polymicrobial biofilms promotes the spread of antibiotic resistance genes (ARGs) (Michaelis and Grohmann 2023). Therefore, the identification and functional characterization of HGT‐acquired genes have become an important direction for understanding microbial ecological adaptation and evolutionary dynamics.

Mangrove ecosystems harbor diverse *Streptomyces* communities that play key roles in ecological balance and the production of bioactive metabolites. Many mangrove‐derived *Streptomyces* strains synthesize pharmacologically active compounds with strong antimicrobial and plant disease‐suppressive activities (Zhou 2022; Zhou et al. 2022; Zhao et al. 2024). Although the genome of *S. sampsonii* from poplar rhizosphere soil has been published (KJ40, NCBI accession number: GCA_001704195.1), the ecological significance of *Streptomyces* in mangrove environments underscores the need to sequence and assemble the genome of *S. sampsonii* from mangrove rhizosphere soil to elucidate its metabolic potential and biocontrol value. In this study, we present a chromosome‐level genome assembly of *S. sampsonii* and perform comprehensive comparative analyses with its close relatives within the genus. By integrating phylogenomics, pangenome profiling, biosynthetic gene cluster (BGC) prediction, and horizontal gene transfer (HGT) detection, we reveal the genomic features that underpin the adaptation and metabolic potential of *S. sampsonii* and related species. These analyses highlight the functional enrichment of pan_shell and pan_cloud genes in xenobiotic biodegradation and antibiotic biosynthesis, the diversity and evolutionary dynamics of BGCs, and the significant contribution of HGT to secondary metabolism. Together, our findings provide new insights into the genomic diversity and ecological roles of *S. sampsonii* and its close relatives, laying a foundation for their future application as biocontrol agents.

## 2 Materials and Methods

### 2.1 Sample Collection and Genome Sequencing

The *Streptomyces sampsonii* (MCCC 1A01650) strain was originally isolated from mangrove rhizosphere soil in the Zhangjiangkou Mangrove National Nature Reserve, Yunxiao County, Fujian Province, China, and was obtained from the Marine Culture Collection of China (MCCC). The strain was first inoculated into Gauze's Synthetic Medium No. 1 and incubated at 28°C for 4 days, followed by transfer into liquid medium in Erlenmeyer flasks and cultured at 28°C with shaking at 140 r/min for 4 days. The mycelia were harvested by centrifugation at 12,000 r/min for 20 min and sent to Genepre Technology Co. Ltd. (Chengdu, China) for whole‐genome sequencing.

The method for library preparation and sequencing is as follows: Qualified DNA (≥ 1.5 μg, concentration ≥ 15 ng/μL, OD260/280 between 1.7 and 2.2) was subjected to SMRTbell library construction using the SMRTbell Express Template Prep Kit 3.0 (PacBio, USA). Briefly, genomic DNA was sheared with g‐TUBE (Covaris, USA), followed by DNA damage repair, end repair/A‐tailing, and ligation of barcoded overhang adapters. After bead‐based purification and size selection, the libraries were quality‐checked with Qubit and Agilent 2100. Sequencing primer and polymerase were bound to the libraries using the PacBio Binding Kit 2.0, and the final complexes were sequenced on the PacBio Sequel II platform with SMRT Cell 8M (PacBio, USA).

### 2.2 Data Acquisition

The reference genome for *S. sampsonii* (GCA_001704195.1) was downloaded from the NCBI Assembly database. To identify closely related *Streptomyces* species, the partial 16S rRNA sequence of *S. sampsonii* ( *S. sampsonii* strain JA8 16S ribosomal RNA gene, partial sequence) was used as a query in an NCBI BLASTn search against the nucleotide (nt) database. Hits with high sequence similarity (≥ 97% nucleotide identity and ≥ 90% query coverage) were considered candidate close relatives. For each candidate, we selected assemblies annotated at the chromosome level in the NCBI Assembly/Genome database and downloaded the complete chromosomal FASTA files. Furthermore, we calculated the ANI and AAI values between *S. sampsonii* and its closely related taxa using FastANI (Jain et al. 2018) and FastAAI (Gerhardt et al. 2025), respectively.

### 2.3 Genome Assembly and Quality Assessment

Raw sequencing data were first processed using fastp (v0.23.4) (Chen 2023) with default parameters for quality control. HiFi reads shorter than 0.5 kb were removed using SeqKit (v2.10.1) (Shen et al. 2016). Genome assembly was then performed with Unicycler (v0.5.1) (Wick et al. 2017) based on the quality‐controlled data. The draft assemblies were further improved and scaffolded against the reference genome using RagTag (v2.1.0) (Alonge et al. 2022). The completeness of the genome assembly was evaluated using BUSCO v6.1.0 (Tegenfeldt et al. 2025) against the actinomycetota_odb12 lineage dataset. Finally, assembly quality was evaluated using QUAST (v5.2.0) (Gurevich et al. 2013).

### 2.4 Genome Annotation and Functional Analysis

Gene structure annotation of all bacterial genomes was performed using Prokka (v1.13) (Seemann 2014). Transfer RNAs (tRNAs) were predicted with tRNAscan‐SE (v2.0) (Chan et al. 2021), and ribosomal RNAs (rRNAs) were annotated using barrnap (v0.9) (https://github.com/tseemann/barrnap). Small nuclear RNAs (snRNAs) and other non‐coding RNAs were annotated against the Rfam database (Griffiths‐Jones et al. 2003). The predicted protein‐coding genes were further functionally annotated using EggNOG‐mapper (v2.1.2) (Cantalapiedra et al. 2021) with the *Streptomyces* database, and Gene Ontology (GO) terms were extracted from the annotation results. KEGG pathway annotation was carried out with KOBAS (v2.0) (Xie et al. 2011). In addition, protein domains were identified using HMMER hmmsearch (v3.4) (Eddy 2023) against the Pfam database. Enrichment analysis was performed using clusterProfiler (Yu et al. 2012), with the Benjamini‐Hochberg (BH) method applied for multiple‐testing correction.

### 2.5 Phylogenomic Analysis

Single‐copy orthologous genes among *S. sampsonii* and its closely related species were identified using OrthoFinder (v2.5.5) (Emms and Kelly 2019). The protein sequences of these single‐copy orthologs were aligned individually with MAFFT v7.526 (Katoh and Standley 2013) and subsequently concatenated to generate a supermatrix for phylogenetic analysis. Based on the concatenated alignment, a maximum‐likelihood (ML) phylogenetic tree was constructed using IQ‐TREE (Nguyen et al. 2015), with branch support assessed by 1000 bootstrap replicates. The resulting phylogenetic tree was finally visualized and annotated using iTOL (https://itol.embl.de/).

### 2.6 Synteny Analysis

*S. sampsonii* and its closely related species (which are reference genomes defined by NCBI) were used for synteny analysis. Coding sequences (CDS) together with their corresponding BED files containing positional information were provided as input to jcvi (Tang et al. 2024) for synteny detection and visualization with default parameters.

### 2.7 Secondary Metabolite Biosynthetic Gene Cluster Prediction

GenBank (GBK) files were used as input for the prediction of secondary metabolite biosynthetic gene clusters (BGCs) in *S. sampsonii* and its closely related species. The analysis was performed using antiSMASH (v7.0) (Medema et al. 2011), with the taxon parameter set to “bacteria”.

### 2.8 Sequence Similarity Network Analysis of BGCs

Sequence similarity network (SSN) analysis of BGCs was performed using BiG‐SCAPE v2.0 (Navarro‐Muñoz et al. 2020). BiG‐SCAPE calculates pairwise distances between BGCs based on Pfam domain composition similarity, which integrates a weighted combination of the Jaccard index, adjacency index, and domain sequence similarity score. Multiple thresholds (‐‐gcf‐cutoffs ranging from 0.05 to 0.95) were tested, and a cutoff value of 0.3 was selected to generate the final sequence similarity network. The resulting networks were visualized using Cytoscape v3.10.4 (Shannon et al. 2003).

### 2.9 Horizontal Gene Transfer (HGT) Identification

HGT events in *S. sampsonii* and its closely related species were identified using HGTector (v2.0b3) (Zhu et al. 2014). The official pre‐built database provided by HGTector was downloaded from the GitHub repository (https://github.com/qiyunlab/HGTector). Protein sequences from each genome were subjected to homology searches using the search module of HGTector with stringent sequence‐similarity thresholds (‐‐evalue 1e‐20 ‐‐identity 30 ‐‐coverage 40) to detect potential homologous genes. To reduce potential false‐positive predictions caused by shared ancestry or ambiguous homology among closely related taxa, the genus Streptomyces (NCBI taxon 1883) was defined as the self group, whereas the family Streptomycetaceae (NCBI taxon 2062) was defined as the close group. The putative donor taxa were further extracted using TaxonKit (Shen and Ren 2021), and the number of genes contributed by each donor was quantified. Finally, the putative taxonomic origins and relative proportions of putative HGT candidates were visualized using KronaTools (Ondov et al. 2015).

### 2.10 Pangenome Construction

The pangenome of *S. sampsonii* and its closely related species was constructed using Roary (Page et al. 2015) with the parameter ‐i 80. Genes were subsequently classified into four categories based on their frequency across the analyzed genomes: (i) core gene cluster, present in 99%–100% of strains; (ii) soft‐core genes, present in more than 95% of strains (≥ 90 strains); (iii) shell gene cluster, present in 15%–95% of strains (15–90 strains); and (iv) cloud gene cluster, present in less than 15% of strains (< 15 strains). The genes within gene clusters were correspondingly defined as core, soft‐core, shell, and cloud genes. To avoid confusion, we use pan_core, pan_soft‐core, pan_cloud, and pan_shell to denote core, soft‐core, cloud, and shell genes in the pangenome, respectively. In addition, pairwise nonsynonymous substitution rates (Ka) and synonymous substitution rates (Ks) among different gene categories were calculated using KaKs_Calculator (v2.1.0) (Wang et al. 2010).

## 3 Results

### 3.1 Genome Assembly and Phylogenetic Analysis of *S. sampsonii*

**FIGURE 1:**
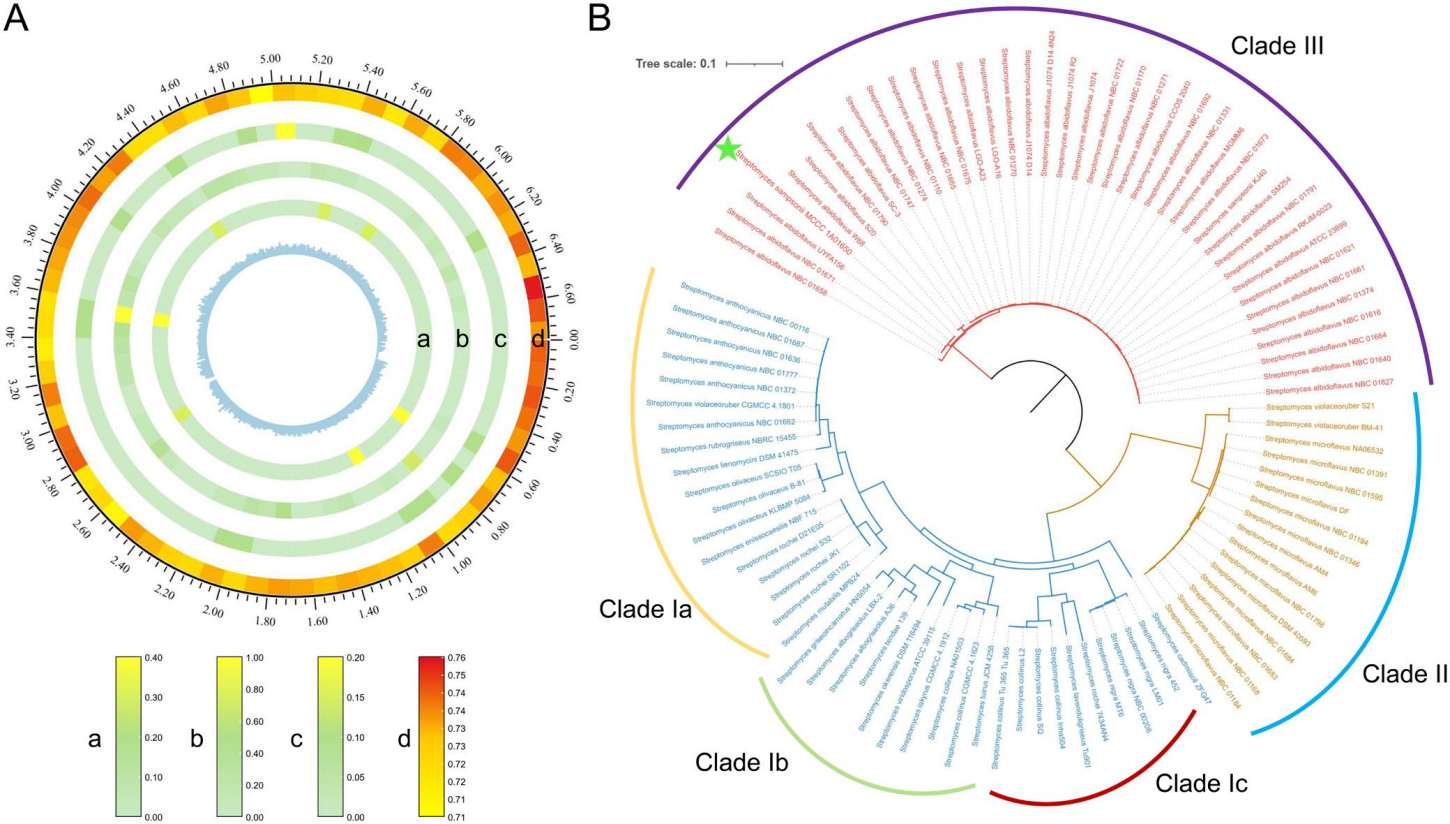
Genome of *S. sampsonii* . (A) Genomic features of *S. sampsonii* . From the inside (a) to the outside (d) are the distributions of protein‐coding genes, tRNAs, rRNAs, sRNAs, and GC content on the chromosome. (B) Phylogenetic tree of *S. sampsonii* and its closely related species. The colors represent different clades.

In this study, we assembled a chromosome‐level genome of *S. sampsonii* MCCC 1A01650. The final assembly had a total size of 7,203,902 bp, comprising a primary chromosome of 6,775,033 bp and 23 additional unplaced contigs that could not be confidently anchored to the chromosome during reference‐guided scaffolding. The assembly quality assessment showed only 43 ambiguous bases (N) per 100 kb, indicating a high‐quality genome. BUSCO assessment showed a completeness of 98.3%, further supporting the high quality and completeness of the genome assembly. The overall GC content was 73.48%, with values ranging between 71% and 76% across 10 kb windows. Genome annotation identified 6015 protein‐coding genes, while noncoding RNA annotation revealed 63 tRNAs, 21 rRNAs, and 16 sRNAs (Figure 1A).

To further investigate the evolutionary relationship between *S. sampsonii* and its closely related species, we collected the genomes of 94 strains from 22 related *Streptomyces* species (Table S1). Phylogenetic analysis divided these strains into three clades (clade I–III) (Figure 1B). Clade I comprised 39 genomes from 19 species. Since *Streptomyces cadmiisoli* ZFG47 was located at the periphery of clade I and did not cluster with any other species, it was assigned as a separate lineage. Excluding *S. cadmiisoli* ZFG47, the remaining species within clade I were further divided into three subgroups: clade Ia, Ib, and Ic. Clade II was mainly composed of strains belonging to *Streptomyces microflavus* and *Streptomyces violaceoruber* , while clade III included two *S. sampsonii* species and 38 *Streptomyces albidoflavus* species. The genome assembled in this study was clustered within clade III. Among all the related species analyzed, *S. sampsonii* was placed in the same evolutionary branch with *S. albidoflavus* , suggesting a close phylogenetic relationship between them. In contrast, the relatively long branch lengths separating clades II and III indicated a more distant relationship between *S. sampsonii* and the species from other clades. In addition, we calculated pairwise Average Nucleotide Identity (ANI) and Average Amino Acid Identity (AAI) values between *S. sampsonii* strains and the analyzed genomes (Figure S1). The results showed that genomes within Clade III exhibited ANI and AAI values above 95% relative to *S. sampsonii* , supporting their assignment to the same species‐level genomic cluster. Genomes from the other clades showed ANI values ranging from 81% to 83% and AAI values ranging from 69% to 72%, indicating clear genomic differentiation from Clade III while remaining within the comparative framework of *S. sampsonii* and its closely related *Streptomyces* taxa. These results provide insights into the genomic features of *S. sampsonii* and clarify its phylogenetic placement within *Streptomyces*.

### 3.2 Chromosomal Synteny Highlights Structural Divergence Among Clades

**FIGURE 2:**
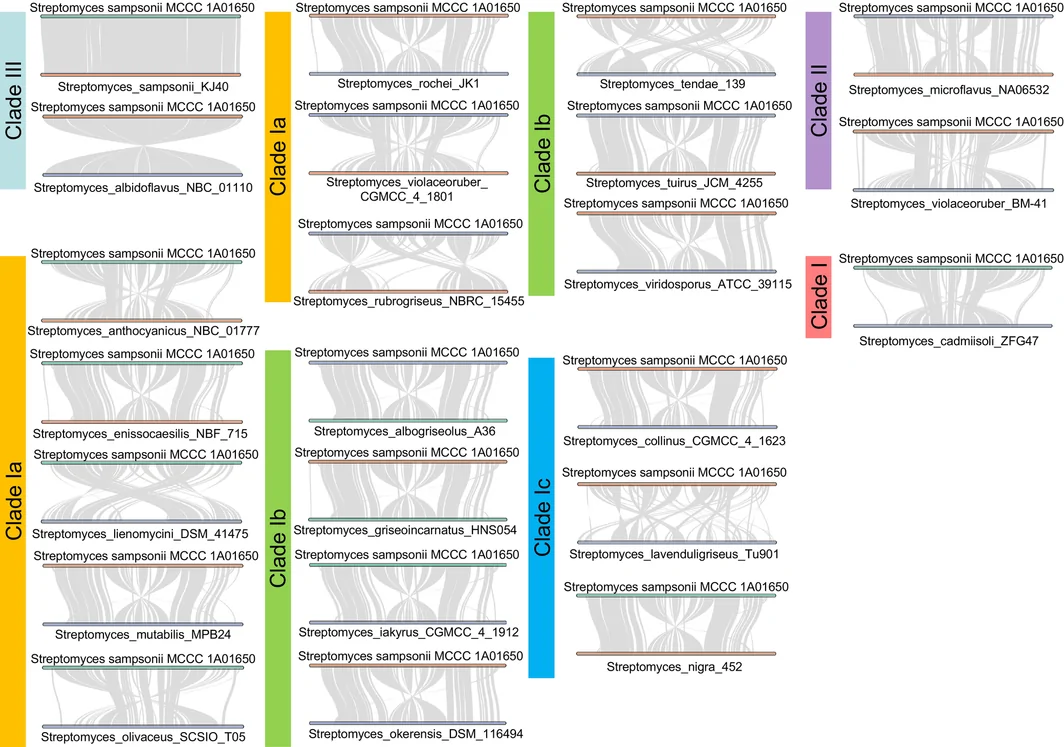
Comparative synteny analysis of *S. sampsonii* and related species. Connecting lines indicate syntenic relationships between chromosomes, and colors represent chromosomes from different strains.

To further compare chromosomal organization across *S. sampsonii* and its closely related *Streptomyces* taxa, we performed a synteny analysis involving representative genomes from different clades. Within Clade III, the analyzed genomes showed a relatively high degree of synteny. Specifically, 5255 and 5311 syntenic genes were identified between *S. sampsonii* MCCC 1A01650 and *S. sampsonii* KJ40, and between *S. sampsonii* MCCC 1A01650 and *S. albidoflavus* , respectively, accounting for 86.9% and 88.3% of the total gene content of *S. sampsonii* MCCC 1A01650. In contrast, comparisons between Clade III and genomes from other clades identified fewer syntenic genes, ranging from 3204 to 3465. Synteny analysis further revealed that large‐scale chromosomal rearrangement patterns, particularly inversion‐like structures, were more frequently observed in comparisons between Clade III and other clades (Figure 2). By comparison, genomes within Clade III exhibited more conserved overall collinearity. These results indicate that chromosomal organization is more similar within Clade III, whereas greater structural divergence is evident between Clade III and the other closely related *Streptomyces* clades. The observed inversion patterns may therefore be associated with the evolutionary divergence between Clade III and these related lineages.

### 3.3 Pangenome of *S. sampsonii* and Its Close Relatives

**FIGURE 3:**
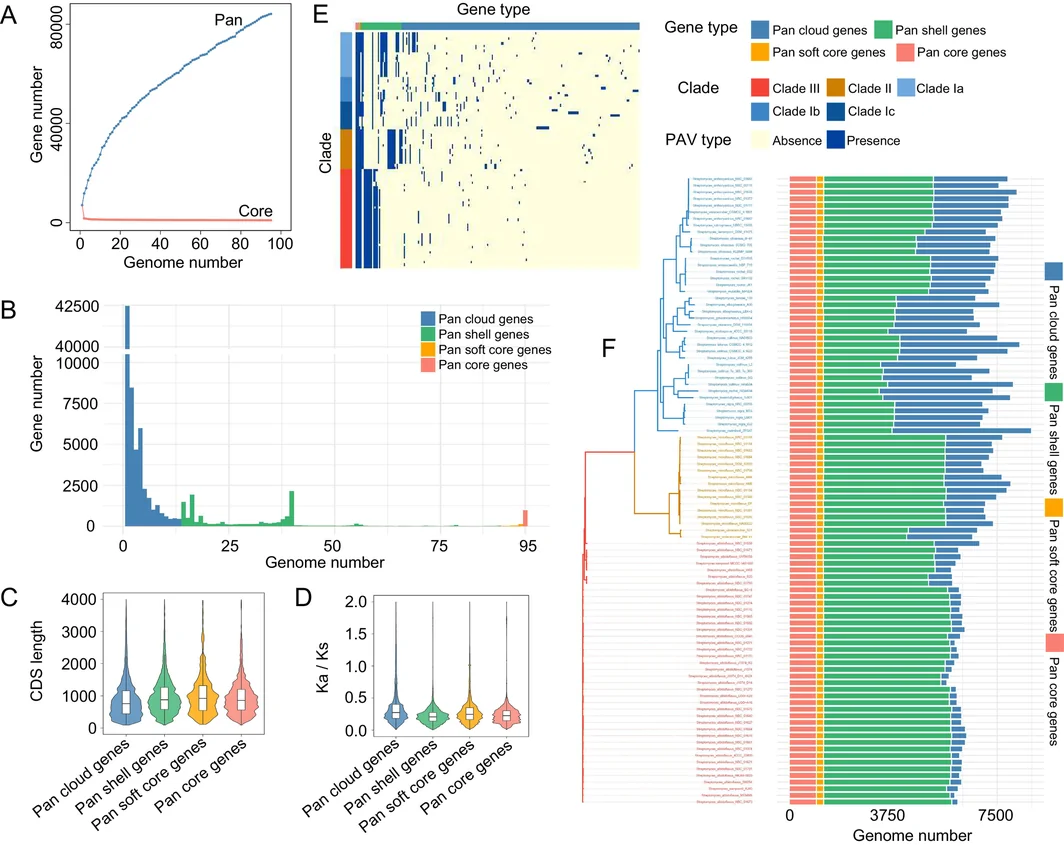
Composition and characteristics of the pan‐genome. (A) Expansion of the total number of gene clusters and reduction of pan_core gene clusters with the inclusion of additional genomes. For each genome number, 100 random combinations were sampled. (B) Number of gene clusters present across different numbers of genomes. (C) CDS length distribution of different types of gene clusters. (D) Distribution of Ka/Ks values among pairwise genes within different types of gene clusters. (E) PAV of genes across different species. (F) Distribution of different types of gene clusters among different species.

To characterize the genomic diversity of *S. sampsonii* and its closely related species, we constructed a pangenome based on protein‐coding genes. The resulting pangenome comprised 84,178 clusters. According to their presence/absence variation (PAV) across species, these clusters were classified into 980 pan_core clusters, 260 pan_soft‐core clusters, 12,661 pan_shell clusters, and 70,677 pan_cloud clusters. With the continuous addition of genomes, the number of core genes tended to stabilize, whereas the total number of genes continued to increase (Figure 3A), indicating that the pangenome of *S. sampsonii* and its relatives is open. Notably, 84% (*n* = 70,677) of the clusters belonged to the pan_cloud cluster, with more than 42,000 clusters found exclusively in a single genome (Figure 3B), suggesting a high level of genetic diversity among the analyzed species. Compared with other gene categories, cloud genes had shorter transcript lengths, with the majority being less than 1000 bp (Figure 3C). In addition, cloud genes exhibited higher Ka/Ks ratios (Figure 3D), implying that they are under higher selection pressure. PAV comparison across different clades further revealed that some pan_shell clusters were restricted to a single clade but absent from others, suggesting that the presence or absence of specific clusters is clade‐related. In particular, 3098 and 3673 clusters were exclusively present in clade II and clade III, respectively, accounting for 55% of all pan_shell clusters (Figure 3E). This indicates that the distribution of these clusters may be shaped by phylogenetic divergence, with clade‐specific patterns of retention or loss. Moreover, strains with closer phylogenetic relationships shared more similar numbers of pan_shell genes, whereas differences in gene content among clades were primarily driven by variation in pan_cloud gene numbers (Figure 3F). Together, these results highlight the complexity and diversity of the *S. sampsonii* genome and those of its close relatives.

### 3.4 Function of Pangenome Gene Categories

**FIGURE 4:**
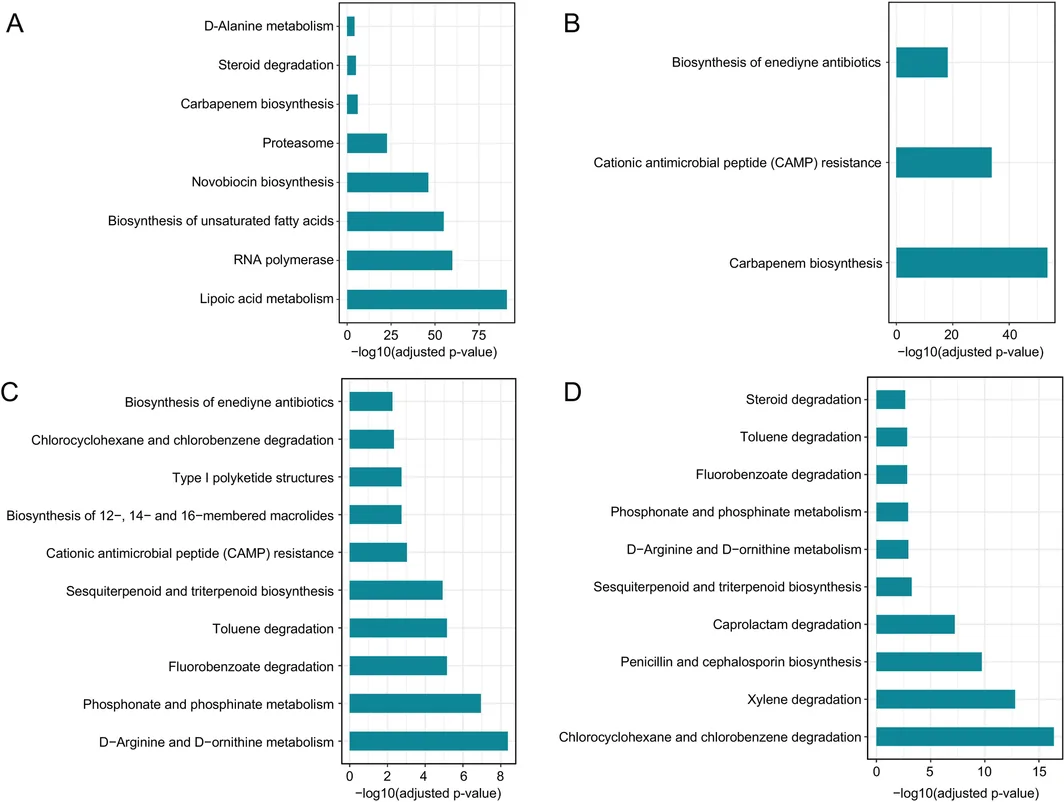
KEGG enrichment analysis of the four gene categories in the pan‐genome. A, B, C, and D represent the enrichment results for pan_core genes, pan_soft‐core genes, pan_shell genes, and pan_cloud genes, respectively.

To further elucidate the functions of pan_core, pan_soft‐core, pan_shell, and pan_cloud genes, KEGG enrichment analysis was performed for these four categories. Pan_core genes were enriched in fundamental metabolic and life activity‐related pathways such as lipoic acid metabolism, RNA polymerase, proteasome, biosynthesis of unsaturated fatty acids, steroid degradation, and D‐alanine metabolism, as well as in antibiotic‐related pathways including novobiocin biosynthesis and carbapenem biosynthesis (Figure 4A). Pan_soft‐core genes were mainly enriched in pathways associated with carbapenem biosynthesis, cationic antimicrobial peptide (CAMP) resistance, and biosynthesis of enediyne antibiotics (Figure 4B). The enrichment of pan_shell genes was largely distributed across three categories of pathways: (i) specialized metabolic pathways, such as D‐arginine and D‐ornithine metabolism and phosphonate and phosphinate metabolism; (ii) xenobiotics biodegradation and metabolism‐related pathways, including fluorobenzoate degradation, chlorocyclohexane and chlorobenzene degradation, and toluene degradation; and (iii) secondary metabolite biosynthesis pathways related to antibiotics, such as sesquiterpenoid and triterpenoid biosynthesis, biosynthesis of 12‐, 14‐, and 16‐membered macrolides, and biosynthesis of enediyne antibiotics (Figure 4). Pan_cloud genes were enriched in 10 pathways, which were mainly concentrated within the same three categories identified for pan_shell genes. Further enrichment analysis of clade‐specific pan_shell genes revealed that these were also predominantly enriched in xenobiotics biodegradation and metabolism and antibiotic biosynthesis pathways, although the enrichment patterns varied among clades: pan_shell genes unique to Clade II and Clade III were mainly enriched in xenobiotics biodegradation and metabolism pathways, whereas those specific to Clade I were enriched in antibiotic biosynthesis‐related pathways such as biosynthesis of Type II polyketide products, polyketide sugar unit biosynthesis, and sesquiterpenoid and triterpenoid biosynthesis (Figure S2). Collectively, these results indicate that the pan_core and pan_soft‐core genes of *S. sampsonii* and its closely related species are primarily involved in maintaining essential cellular functions and antibiotic biosynthesis, whereas pan_shell and pan_cloud genes may contribute to genomic diversity and environmental adaptation.

### 3.5 BGCs in *S. sampsonii* and Its Closely Related Species

**FIGURE 5:**
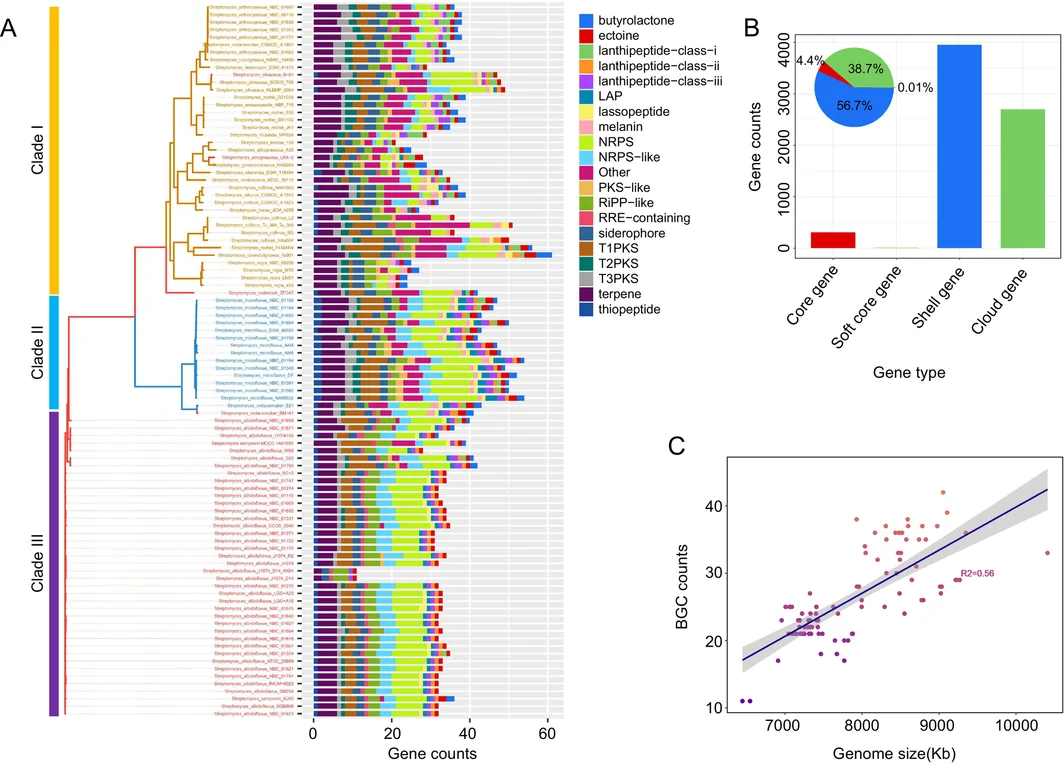
Secondary metabolite BGCs in *S. sampsonii* and its related species. (A) Distribution of BGC types in *S. sampsonii* and closely related species. (B) PAV patterns of BGC_core genes across the pangenome. (C) Correlation analysis between the number of BGCs and genome size.

Given that *S. sampsonii* and its closely related species have the potential to synthesize antibiotics and other secondary metabolites, this study annotated the BGCs of *S. sampsonii* and its closely related species. A total of 2516 secondary metabolite BGCs were identified, which are predicted to be associated with the biosynthesis of 48 classes of secondary metabolites. On average, each strain harbored 26.5 BGCs. Some BGCs were predicted to have the potential to contribute to the biosynthesis of more than one type of secondary metabolite (Table S2). For instance, the CP140156.1.region001 gene cluster of *S. albidoflavus* ATCC 23899 was predicted to encode the biosynthetic potential for not only NRPS but also terpene. In addition, the proportions of different types of BGCs varied. Among them, five classes, such as NRPS, terpene, T1PKS, NRPS‐like, and RiPP‐like, together accounted for more than 50% of the total BGCs, indicating that *S. sampsonii* and its close relatives exhibit distinct patterns in their secondary metabolite biosynthetic potential. Notably, several identified BGC classes are commonly associated with biocontrol‐related traits, including NRPS and PKS clusters potentially involved in the biosynthesis of antimicrobial compounds, siderophore clusters potentially involved in iron acquisition and competition, and RiPP‐related clusters predicted to encode antimicrobial peptide‐like compounds. Moreover, the distribution of these BGCs differed across clades, with species within the same clade tending to share similar BGC profiles and biosynthetic potentials. Some BGC types associated with specific metabolite classes were even restricted to species from particular clades. For example, melanin and butyrolactone were mainly produced by species in clades I and II, whereas RE‐containing metabolites were primarily BGCs were primarily detected in species from clade III (Figure 5A).

According to antiSMASH, the key genes directly encoding metabolites within BGCs are referred to as core genes, which are designated as BGC_core genes in this study to distinguish them from pangenome core genes. Among the identified BGCs, 56.7% of the BGC_core genes belonged to pan_shell genes, while 38.7% were classified as pan_cloud genes (Figure 5B), suggesting that these BGC_core genes often fall into the variable gene category (not pan_core genes) of the pangenome. This implies that the origins of these BGCs may be diverse. Furthermore, the number of secondary metabolite BGCs was correlated with genome size (*R* ^2^ = 0.56), suggesting that genome size may be one of the driving factors for the variation in BGC abundance (Figure 5C). Collectively, these findings provide a foundation for understanding the metabolic diversity and evolutionary dynamics of *S. sampsonii* and its closely related species.

### 3.6 Sequence Similarity Network Analysis

**FIGURE 6:**
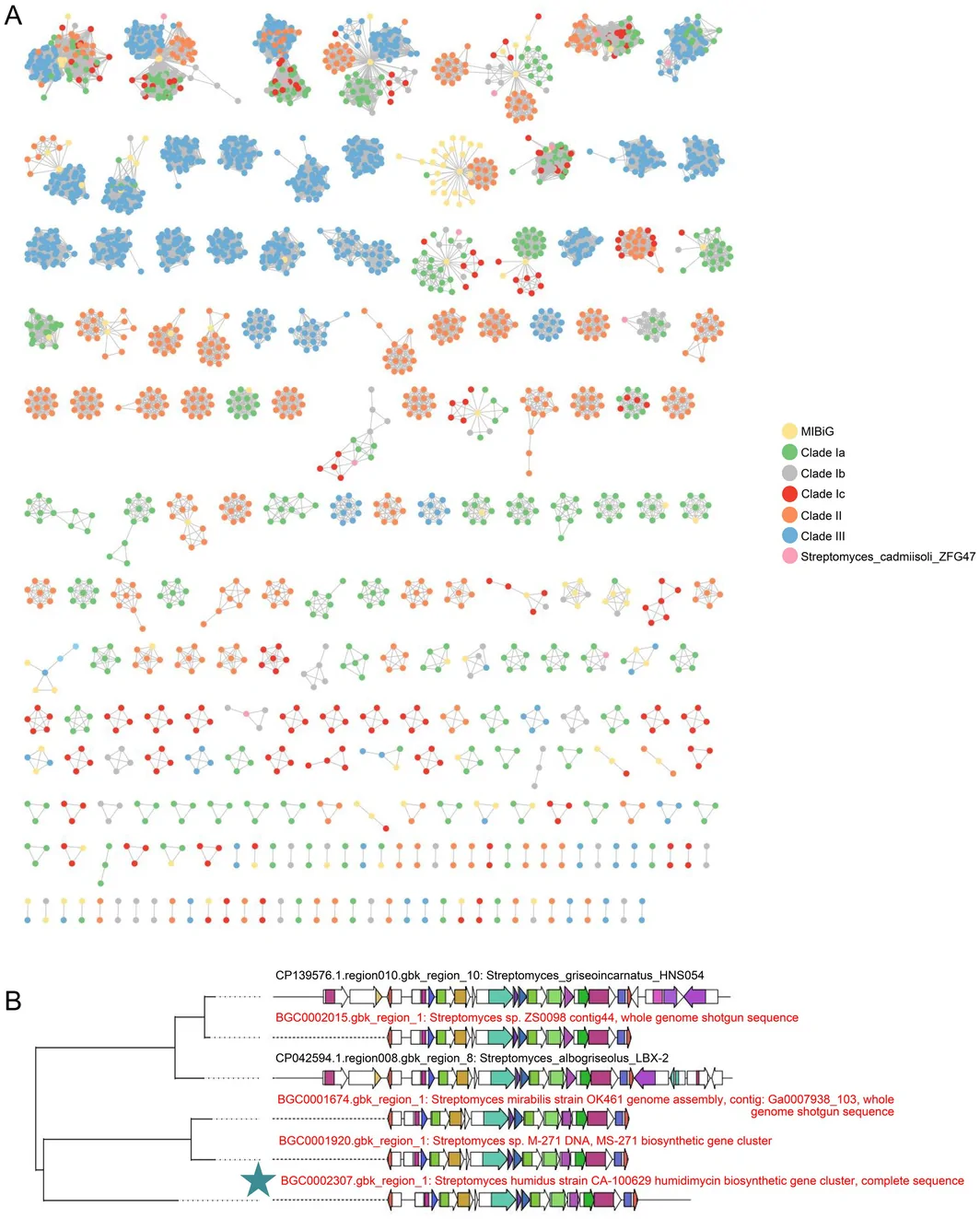
Functional analysis of BGCs using a sequence similarity network. (A) BiG‐SCAPE similarity network, where each node represents a single BGC and edges indicate similarity relationships between BGCs. Yellow nodes indicate reference BGCs, while nodes in other colors represent BGCs from species belonging to different clades. (B) Schematic representation of gene cluster structures within GCF FAM_00398. The text above each cluster indicates the corresponding cluster ID. Reference BGCs are shown in red, with the humidimycin biosynthetic reference BGC highlighted by a star.

To further elucidate the functions of these gene clusters, a similarity network was constructed using experimentally validated metabolite BGCs from the MIBiG database together with the BGCs identified in this study. Apart from 325 BGCs that did not cluster into any gene cluster families (GCFs), the remaining 2300 BGCs were grouped into 256 GCFs by the similarity network (Figure 6A, Table S3). Among them, 232 GCFs were exclusively composed of BGCs from species in a single clade, suggesting that the formation of these GCFs may be associated with species evolution. Notably, 59 of the 256 GCFs contained reference BGCs from the MIBiG database, allowing functional inference of these GCFs. For instance, in the GCF designated FAM_00398, BGCs from *Streptomyces griseoincarnatus* and *Streptomyces albogriseolus* showed high conservation in domain architecture and transcriptional orientation with the known humidimycin biosynthetic gene cluster BGC0002307, indicating that these BGCs are highly likely to possess the potential to synthesize humidimycin or its analogs (Figure 6B). In addition, 1936 BGCs annotated in this study (76.9%) were grouped into GCFs without any reference BGCs, implying that the majority of BGC functions remain unknown and warrant further investigation.

### 3.7 HGT in *S. sampsonii* and Its Closely Related Species

**FIGURE 7:**
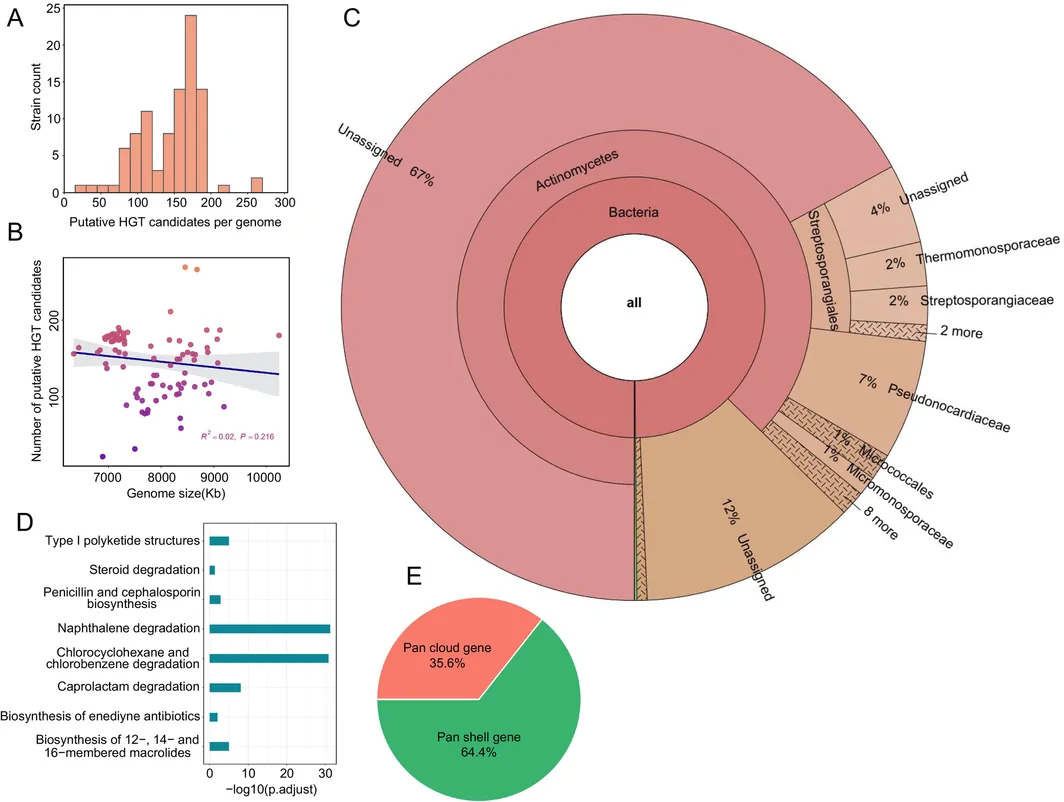
Putative HGT candidates in *S. sampsonii* and its closely related species. (A) Distribution of the number of putative HGT candidates. (B) Correlation between the number of putative HGT candidates and genome size. (C) Putative donor of putative HGT candidates. (D) KEGG enrichment analysis of putative HGT candidates. (E) Classification of putative HGT candidates in the pangenome.

HGT is a major driver of bacterial evolution, enabling rapid adaptation to changing environments. In this study, we identified HGT events in *S. sampsonii* and its closely related species. From 95 genomes, 13,996 putative HGT candidates were identified, with an average of 147.3 per genome (Table S4). *Streptomyces collinus* L2 and SQ carried the fewest (22 and 32), while *Streptomyces olivaceus* SCSIO T05 had the most (269) (Figure 7A). The number of putative HGT candidates showed no significant correlation with genome size (*R* ^2^ = 0.02, *p* = 0.216) (Figure 7B), suggesting that variation in candidate number was not primarily explained by genome size. To further evaluate the reliability of the predicted HGT candidates, we compared GC content (Figure S3) and codon usage (Figure S4) between putative HGT candidates and non‐HGT genes within each genome. Overall, the putative HGT candidates showed slightly lower total GC, GC1, and GC2 values and slightly higher GC3 values than non‐HGT genes, together with reproducible shifts in the relative usage of several synonymous codons. These compositional differences provide additional support for the reliability of the HGT predictions. However, the magnitude of these differences varied among genomes, and some individual GC or codon‐usage indices showed little or no difference between the two gene sets. This is not unexpected because GC content and codon usage represent supporting rather than definitive indicators of HGT. Horizontally acquired genes may gradually become compositionally similar to the recipient genome through amelioration, while transfers between closely related taxa with similar genomic composition may exhibit only weak compositional differences. In addition, analysis of the best‐matching taxonomic groups showed that over 87% of putative HGT candidates were most closely associated with Actinomycetota, with 11%, 8% and 2% of Actinomycetes derived from Streptosporangiales, Pseudonocardiaceae, and Micrococcales (Figure 7C), suggesting that these groups represent major putative donor‐related lineages for the inferred horizontal transfer signals.

KEGG enrichment showed that putative HGT candidates were mainly involved in xenobiotic degradation pathways (e.g., naphthalene degradation, chlorocyclohexane and chlorobenzene degradation) and antibiotic biosynthesis pathways (e.g., 12‐, 14‐, and 16‐membered macrolides; Type I polyketides) (Figure 7D). These results are consistent with the enrichment patterns observed in pan_shell and pan_cloud genes of the pangenome. Similarly, 64.4% (*n* = 4987) of putative HGT candidates were pan_shell genes, and 35.6% (*n* = 9008) were pan_cloud genes in the pangenome (Figure 7E), suggesting HGT may be an important source of variable genes. Furthermore, 148 BGC_core genes were identified among the putative HGT candidates, representing 14 predicted metabolite classes (Table S5). The most abundant were T1PKS (*n* = 49), betalactone (*n* = 19) and NRPS (*n* = 17), indicating that *S. sampsonii* and related species may have acquired the capacity to produce diverse secondary metabolites via horizontal gene transfer.

## 4 Discussion

High‐quality genome assemblies provide a valuable foundation for exploring species evolutionary relationships and genomic diversity. As of October 9, 2025, a total of 12,592 *Streptomyces* genomes have been recorded in the NCBI database. Members of the *Streptomyces* genus typically possess large, GC‐rich genomes ranging from 6.7 to 10.1 Mb, generally composed of a single linear chromosome (Lee, Kim, Hwang, et al. 2020). Consistent with the general features of *Streptomyces*, the assembled genome of *S. sampsonii* in this study is 7.20 Mb in size, with an overall GC content of 73.48%. The relatively high GC content and compact genomic structure are typical characteristics of *Streptomyces* genomes, supporting their metabolic diversity and environmental adaptability (Wright and Bibb 1992). Furthermore, phylogenetic, ANI, and AAI analyses consistently supported the clustering of the Clade III genomes within the same species‐level genomic group. Consistent with this relationship, genomes within Clade III exhibited a relatively high degree of chromosomal synteny and conserved overall collinearity. In contrast, more pronounced differences in chromosomal organization were observed between Clade III and the other clades included in the comparative dataset. These results suggest that chromosomal structural variation is associated with the evolutionary differentiation among *S. sampsonii* and its closely related Streptomyces taxa. It is worth noting that reference‐guided scaffolding with RagTag may partially mask strain‐specific structural variations by constraining contig ordering and orientation according to the reference genome, and this potential bias should therefore be considered when interpreting chromosomal structural differences. In contrast, *S. sampsonii* exhibits chromosomal inversions when compared with more distantly related clades, consistent with earlier observations that large‐scale translocations and rearrangements frequently occur among different *Streptomyces* subgroups (Yoon‐Hee et al. 2021). These results suggest that closely related species maintain high structural stability, while chromosomal rearrangements across clades may represent a major driver of interclade divergence.

Extensive studies on bacterial pangenomes have revealed diverse evolutionary patterns across species, with a considerable proportion exhibiting an open pangenome structure. For example, the *Escherichia coli* pangenome comprises over 13,000 genes and continues to expand as more genomes are sequenced (Rasko et al. 2008); similarly, the pangenome of the genus *Pseudoalteromonas* increases with the addition of new genomes, with both the genus as a whole and specific subgroups (such as the pigmented strains group) showing open characteristics (Bosi et al. 2017). In addition, the *Corynebacterium striatum* pangenome has also been demonstrated to be open, with new variable genes continuously identified as sample size increases (Nageeb and Hetta 2023). These findings indicate that pangenome openness is a common phenomenon among bacterial groups with broad ecological distribution, frequent gene flow, or diverse metabolic capacities. In this study, the pangenome of *S. sampsonii* and its closely related species was also found to be open, suggesting that even within a relatively narrow phylogenetic range, actinomycete populations maintain a high potential for genetic innovation, consistent with the evolutionary patterns observed in other open‐type bacterial pangenomes. In this study, the pangenome of *S. sampsonii* and its closely related *Streptomyces* taxa also exhibited an open structure, characterized by a large proportion of accessory, pan_cloud, and pan_private genes. This pattern is consistent with previous studies of *Streptomyces*, which have reported extensive genomic diversity and highly variable gene repertoires (Caicedo‐Montoya et al. 2021; Passari et al. 2025). The open pangenome structure observed here may reflect the high genomic plasticity of *Streptomyces* populations and their long‐term evolutionary diversification. Previous genus‐level and *Streptomyces* pangenome studies have used Roary with reduced protein‐identity thresholds to accommodate sequence divergence among related taxa (Caicedo‐Montoya et al. 2021; Mustapha et al. 2022). Nevertheless, such clustering settings may influence ortholog grouping and increase the apparent proportion of pan_cloud and pan_private genes. At the same time, we also acknowledge that the observed proportion of pan_cloud and pan_private genes may also be influenced by genome sampling and the availability of reference resources. Since this study was limited to chromosome‐level genomes, the current dataset may not fully represent the total genetic diversity of *Streptomyces*, potentially contributing to the identification of a large number of lineage‐specific genes. Therefore, the open pangenome pattern observed in *S. sampsonii* and related *Streptomyces* taxa likely results from the combined effects of biological diversification and the current availability of genomic resources.

The openness of a pangenome is typically driven by multiple mechanisms: frequent gene acquisition, particularly through HGT and mobile genetic elements, continuously introduces new functions into the gene pool; selective pressures arising from different ecological niches and lifestyles (generalists vs. specialists) maintain a dynamic balance between gene gain and loss; and large genomes with abundant BGCs provide a potential reservoir for variable gene accumulation (McInerney et al. 2017; Dewar et al. 2024). Previous studies have shown that *Streptomyces* genomes exhibit remarkable plasticity and strong signatures of HGT (Abdoul‐Razak et al. 2019), which may represent a key driving force behind their open pangenome structure. Consistent with these observations, our study found that the majority (95.4%) of BGC_core genes within secondary metabolite BGCs belong to the pan_shell and pan_cloud categories, indicating that secondary metabolite‐related genes are highly variable and evolutionarily dynamic. Notably, no melanin biosynthetic gene clusters were detected in Clade III, where the colonies predominantly exhibit golden or yellow pigmentation and lack black‐colored strains, which is consistent with the loss of melanin‐related metabolic capacity. This suggests that the diversity of secondary metabolites is an important manifestation of pangenome openness. Moreover, all putative HGT candidates were classified as pan_shell or pan_cloud genes, suggesting that predicted horizontal transfer signals are concentrated within the variable component of the pangenome. Despite the overall high proportion of pan_shell and pan_cloud genes in the pangenome (84%), the number of putative HGT candidates showed no significant correlation with genome size, suggesting that the distribution of these variable genes is not simply determined by genome size and may instead reflect lineage‐specific gene acquisition and diversification. From a broader perspective, *S. sampsonii* is an actinomycete species with great potential for biological control. The rich and diverse metabolism‐related genes identified in *S. sampsonii* and its close relatives provide clues for discovering bioactive metabolites with novel structures and functions. For instance, in this study, pan_shell genes associated with antibiotic biosynthesis were present in Clade I *Streptomyces* species but absent in Clade III, suggesting that Clade I species may possess the capacity to synthesize specific antibiotics. These genes and their corresponding metabolic products warrant further functional exploration. However, it should be noted that the BGCs and HGT events identified in this study are based on computational predictions and therefore require further experimental validation.

It should be noted that the openness of the pangenome is influenced not only by biological characteristics but also by sample selection and the comparative scale applied. Compared with broad‐scale analyses across genera or families, pangenome studies focusing on closely related species or intra‐species populations can reveal fine‐scale evolutionary events with higher phylogenetic resolution. For example, comparative studies between highly related *Streptomyces* species have shown that while their pan_core genomic structures are highly conserved, genes related to secondary metabolism and regulatory or signal transduction pathways exhibit marked divergence. This indicates that ecological and functional differentiation continues to occur even among closely related *Streptomyces* species (Nett et al. 2009). Similarly, the pangenome constructed for *S. sampsonii* and its close relatives revealed gene gain and loss events over a short evolutionary timescale, particularly involving pan_shell and pan_cloud genes enriched in pathways related to xenobiotic degradation, antibiotic biosynthesis, and defense mechanisms. These findings not only demonstrate the rapid ecological and metabolic diversification within closely related *Streptomyces* populations but also highlight the unique value of pangenome analysis within narrow taxonomic groups for elucidating microevolutionary and functional innovation processes. In addition, *S. sampsonii* is a promising biocontrol actinomycete, and its ecological competitiveness and stress tolerance in soil environments are closely associated with genomic plasticity. Previous studies have suggested that *Streptomyces* species continuously remodel their secondary metabolite biosynthetic gene clusters to adapt to complex microbial competition and to produce diverse antibiotics (Lee, Kim, Chung, et al. 2020; Qi et al. 2021; Westhoff et al. 2021). Consistently, this study found that a large proportion of BGC_core genes belong to the pan_shell and pan_cloud in the pangenome, further supporting the role of secondary metabolism as a key driver of genomic plasticity in *S. sampsonii* populations. On the other hand, several BGC classes identified in this study have been previously linked to biocontrol‐associated traits. For example, NRPS and PKS clusters are commonly involved in the biosynthesis of antimicrobial compounds, while siderophore‐associated clusters may facilitate microbial competition through iron acquisition. In addition, RiPP‐like and lanthipeptide clusters may contribute to the production of antimicrobial peptides. The presence and diversification of these BGCs may partially explain the reported biocontrol activities of some *S. sampsonii* and its closely related species. Moreover, the significant enrichment of pan_shell and pan_cloud genes in the “xenobiotics biodegradation and metabolism” pathway suggests that these strains may have acquired or retained specific variant genes to enhance tolerance and degradation capabilities toward pesticides, solvents, and other anthropogenic organic compounds. A considerable fraction of these environment‐adaptive genes were identified among the putative HGT candidates, with most showing best‐matching taxonomic associations with other Actinomycetota lineages. The enrichment of putative HGT candidates in “xenobiotics biodegradation and metabolism” and antibiotic biosynthesis pathways suggests that horizontally transferred genes may have contributed to the diversification of metabolic functions and, consequently, to the ecological adaptability and competitive potential of *S. sampsonii* and its closely related taxa. Collectively, these findings deepen our understanding of the mechanisms underlying ecological adaptation and metabolic evolution in actinomycetes and provide a theoretical and genomic foundation for developing efficient and environmentally friendly biocontrol resources.

## Author Contributions

**Boyang Zhang:** conceptualization (equal), data curation (equal), funding acquisition (equal), methodology (equal), project administration (equal), software (equal), supervision (equal), validation (equal), writing – original draft (equal), writing – review and editing (equal). **Shuo Ji:** formal analysis (equal), methodology (equal), software (equal), validation (equal), writing – original draft (equal).

## Funding

This research was funded by Science Research Project of Hebei Education Department (Grant number BJK2023104).

## Conflicts of Interest

The authors declare no conflicts of interest.

## Supporting information


**Figure S1:** Genome‐wide similarity analysis between *S. sampsonii* (KJ40 and MCCC 1A01650) and related *Streptomyces* taxa based on ANI (right) and AAI (left) values.

**Figure S2:** KEGG enrichment results of pan_core, pan_soft‐core, pan_private, and branch‐specific pan_shell genes.

**Figure S3:** Genome‐paired differences in total GC, GC1, GC2 and GC3, calculated as HGT minus non‐HGT values in percentage points. Gray points represent individual genomes; diamonds and error bars indicate mean differences and 95% confidence intervals.

**Figure S4:** Genome‐paired differences in relative synonymous codon usage (RSCU) for multi‐codon amino‐acid families. Gray points represent individual genomes, diamonds indicate mean HGT‐minus‐non‐HGT differences, and horizontal lines denote 95% confidence intervals. Black dots indicate Benjamini–Hochberg‐adjusted (*q* < 0.05) across all 59 comparisons.

**Table S1:** Genome assemblies of close relatives of *S. sampsonii* analyzed in this study.

**Table S2:** Summary of BGCs across the *S. sampsonii* clade.

**Table S3:** Results of the BiG‐SCAPE similarity network analysis of BGCs.

**Table S4:** Putative HGT candidates identified in this study.

**Table S5:** Putative HGT candidates shared with core genes of BGCs.

## Data Availability

Raw Data of *Streptomyces sampsonii* and the assembly as well as annotation files of *S. sampsonii* can be downloaded from the figshare (https://figshare.com/articles/dataset/_b_Raw_data_of_b_b_Streptomyces_sampsonii_b_/30577256?file=59424803).

## References

[ece374350-bib-0001] Abdoul‐Razak, T. , L. Jean‐Noël , T. Maxime , et al. 2019. “Massive Gene Flux Drives Genome Diversity Between Sympatric Streptomyces Conspecifics.” MBio 10: e01533‐19. 10.1128/mbio.01533-19. 31481382 PMC6722414

[ece374350-bib-0002] Alonge, M. , L. Lebeigle , M. Kirsche , et al. 2022. “Automated Assembly Scaffolding Using RagTag Elevates a New Tomato System for High‐Throughput Genome Editing.” Genome Biology 23: 258. 10.1186/s13059-022-02823-7. 36522651 PMC9753292

[ece374350-bib-0003] Annabelle, T. , and L. Pierre . 2016. “Complete Genome Sequence of *Streptomyces ambofaciens* DSM 40697, a Paradigm for Genome Plasticity Studies.” Genome Announcements 4: e00470‐16. 10.1128/genomea.00470-16. 27257195 PMC4891641

[ece374350-bib-0004] Arnold, B. J. , I.‐T. Huang , and W. P. Hanage . 2022. “Horizontal Gene Transfer and Adaptive Evolution in Bacteria.” Nature Reviews. Microbiology 20: 206–218. 10.1038/s41579-021-00650-4. 34773098

[ece374350-bib-0005] Bentley, S. D. , K. F. Chater , A.‐M. Cerdeño‐Tárraga , et al. 2002. “Complete Genome Sequence of the Model Actinomycete *Streptomyces coelicolor* A3(2).” Nature 417: 141–147. 10.1038/417141a. 12000953

[ece374350-bib-0006] Bosi, E. , M. Fondi , V. Orlandini , et al. 2017. “The Pangenome of (Antarctic) Pseudoalteromonas Bacteria: Evolutionary and Functional Insights.” BMC Genomics 18: 93. 10.1186/s12864-016-3382-y. 28095778 PMC5240218

[ece374350-bib-0007] Caicedo‐Montoya, C. , M. Manzo‐Ruiz , and R. Ríos‐Estepa . 2021. “Pan‐Genome of the Genus Streptomyces and Prioritization of Biosynthetic Gene Clusters With Potential to Produce Antibiotic Compounds.” Frontiers in Microbiology 12: 677558. 10.3389/fmicb.2021.677558. 34659136 PMC8510958

[ece374350-bib-0008] Cantalapiedra, C. P. , A. Hernández‐Plaza , I. Letunic , P. Bork , and J. Huerta‐Cepas . 2021. “eggNOG‐mapper v2: Functional Annotation, Orthology Assignments, and Domain Prediction at the Metagenomic Scale.” Molecular Biology and Evolution 38: 5825–5829. 10.1093/molbev/msab293. 34597405 PMC8662613

[ece374350-bib-0009] Chan, P. P. , B. Y. Lin , A. J. Mak , and T. M. Lowe . 2021. “tRNAscan‐SE 2.0: Improved Detection and Functional Classification of Transfer RNA Genes.” Nucleic Acids Research 49: 9077–9096. 10.1093/nar/gkab688. 34417604 PMC8450103

[ece374350-bib-0010] Chen, S. 2023. “Ultrafast One‐Pass FASTQ Data Preprocessing, Quality Control, and Deduplication Using Fastp.” iMeta 2: e107. 10.1002/imt2.107. 38868435 PMC10989850

[ece374350-bib-0011] Deng, Y. , H. Xu , Y. Su , et al. 2019. “Horizontal Gene Transfer Contributes to Virulence and Antibiotic Resistance of *Vibrio harveyi* 345 Based on Complete Genome Sequence Analysis.” BMC Genomics 20: 761. 10.1186/s12864-019-6137-8. 31640552 PMC6805501

[ece374350-bib-0012] Dewar, A. E. , C. Hao , L. J. Belcher , M. Ghoul , and S. A. West . 2024. “Bacterial Lifestyle Shapes Pangenomes.” Proceedings of the National Academy of Sciences of the United States of America 121: e2320170121. 10.1073/pnas.2320170121. 38743630 PMC11126918

[ece374350-bib-0013] Domman, D. , M.‐L. Quilici , M. J. Dorman , et al. 2017. “Integrated View of *Vibrio cholerae* in the Americas.” Science 358: 789–793. 10.1126/science.aao2136. 29123068

[ece374350-bib-0014] Doron, S. , S. Melamed , G. Ofir , et al. 2018. “Systematic Discovery of Antiphage Defense Systems in the Microbial Pangenome.” Science 359: eaar4120. 10.1126/science.aar4120. 29371424 PMC6387622

[ece374350-bib-0015] Eddy, S. R. 2023. “HMMER: Biosequence Analysis Using Profile Hidden Markov Models.” http://hmmer.org/.

[ece374350-bib-0016] Emms, D. M. , and S. Kelly . 2019. “OrthoFinder: Phylogenetic Orthology Inference for Comparative Genomics.” Genome Biology 20: 1–14. 10.1186/s13059-019-1832-y. 31727128 PMC6857279

[ece374350-bib-0017] Gerhardt, K. , C. A. Ruiz‐Perez , L. M. Rodriguez‐R , et al. 2025. “FastAAI: Efficient Estimation of Genome Average Amino Acid Identity and Phylum‐Level Relationships Using Tetramers of Universal Proteins.” Nucleic Acids Research 53: gkaf348. 10.1093/nar/gkaf348. 40287826 PMC12034039

[ece374350-bib-0018] Griffiths‐Jones, S. , A. Bateman , M. Marshall , A. Khanna , and S. R. Eddy . 2003. “Rfam: An RNA Family Database.” Nucleic Acids Research 31: 439–441. 10.1093/nar/gkg006. 12520045 PMC165453

[ece374350-bib-0019] Gurevich, A. , V. Saveliev , N. Vyahhi , and G. Tesler . 2013. “QUAST: Quality Assessment Tool for Genome Assemblies.” Bioinformatics 29: 1072–1075. 10.1093/bioinformatics/btt086. 23422339 PMC3624806

[ece374350-bib-0020] Hodgson, D. A. 2000. “Primary Metabolism and Its Control in Streptomycetes: A Most Unusual Group of Bacteria.” Advances in Microbial Physiology 42: 47–238. 10.1016/S0065-2911(00)42003-5. 10907551

[ece374350-bib-0021] Jain, C. , L. M. Rodriguez‐R , A. M. Phillippy , K. T. Konstantinidis , and S. Aluru . 2018. “High Throughput ANI Analysis of 90K Prokaryotic Genomes Reveals Clear Species Boundaries.” Nature Communications 9: 5114. 10.1038/s41467-018-07641-9. PMC6269478 30504855

[ece374350-bib-0022] Jain, P. K. , and P. C. Jain . 2007. “Isolation, Characterization and Antifungal Activity of *Streptomyces sampsonii* GS 1322.” Indian Journal of Experimental Biology 45: 203–206. http://europepmc.org/abstract/MED/17375561. 17375561

[ece374350-bib-0023] Katoh, K. , and D. M. Standley . 2013. “MAFFT Multiple Sequence Alignment Software Version 7: Improvements in Performance and Usability.” Molecular Biology and Evolution 30: 772–780. 10.1093/molbev/mst010. 23329690 PMC3603318

[ece374350-bib-0024] Kim, S.‐S. , S.‐I. Kang , J.‐S. Kim , et al. 2011. “Biological Control of Root‐Knot Nematode by *Streptomyces sampsonii* KK1024.” Korean Journal of Soil Science and Fertilizer 44, no. 6: 1150–1157.

[ece374350-bib-0025] Lee, N. , W. Kim , J. Chung , et al. 2020. “Iron Competition Triggers Antibiotic Biosynthesis in *Streptomyces coelicolor* During Coculture With *Myxococcus xanthus* .” ISME Journal 14: 1111–1124. 10.1038/s41396-020-0594-6. 31992858 PMC7174319

[ece374350-bib-0026] Lee, N. , W. Kim , S. Hwang , et al. 2020. “Thirty Complete Streptomyces Genome Sequences for Mining Novel Secondary Metabolite Biosynthetic Gene Clusters.” Scientific Data 7: 55. 10.1038/s41597-020-0395-9. 32054853 PMC7018776

[ece374350-bib-0027] Li, S. , T. Zhu , Y. Peng , M. Lei , and S. Han . 2014. “Characteristics of Chitinase‐Produced by Streptomycete Sampsonii With Antimicrobial Activity and Its Biocontrol to Rhizoctonia Violacea.” Journal of Northeast Forestry University 42: 116–121.

[ece374350-bib-0028] Malviya, M. K. , A. Pandey , P. Trivedi , G. Gupta , and B. Kumar . 2009. “Chitinolytic Activity of Cold Tolerant Antagonistic Species of Streptomyces Isolated From Glacial Sites of Indian Himalaya.” Current Microbiology 59: 502–508. 10.1007/s00284-009-9466-z. 19688382

[ece374350-bib-0029] McInerney, J. O. , A. McNally , and M. J. O'Connell . 2017. “Why Prokaryotes Have Pangenomes.” Nature Microbiology 2: 17040. 10.1038/nmicrobiol.2017.40. 28350002

[ece374350-bib-0030] Medema, M. H. , K. Blin , P. Cimermancic , et al. 2011. “antiSMASH: Rapid Identification, Annotation and Analysis of Secondary Metabolite Biosynthesis Gene Clusters in Bacterial and Fungal Genome Sequences.” Nucleic Acids Research 39: W339–W346. 10.1093/nar/gkr466. 21672958 PMC3125804

[ece374350-bib-0031] Michaelis, C. , and E. Grohmann . 2023. “Horizontal Gene Transfer of Antibiotic Resistance Genes in Biofilms.” Antibiotics 12: 328. 10.3390/antibiotics12020328. 36830238 PMC9952180

[ece374350-bib-0032] Mustapha, M. M. , V. R. Srinivasa , M. P. Griffith , et al. 2022. “Genomic Diversity of Hospital‐Acquired Infections Revealed Through Prospective Whole‐Genome Sequencing‐Based Surveillance.” mSystems 7: e01384‐21. 10.1128/msystems.01384-21. 35695507 PMC9238379

[ece374350-bib-0033] Nageeb, W. M. , and H. F. Hetta . 2023. “Pangenome Analysis of *Corynebacterium striatum* : Insights Into a Neglected Multidrug‐Resistant Pathogen.” BMC Microbiology 23: 252. 10.1186/s12866-023-02996-6. 37684624 PMC10486106

[ece374350-bib-0034] Navarro‐Muñoz, J. C. , N. Selem‐Mojica , M. W. Mullowney , et al. 2020. “A Computational Framework to Explore Large‐Scale Biosynthetic Diversity.” Nature Chemical Biology 16: 60–68. 10.1038/s41589-019-0400-9. 31768033 PMC6917865

[ece374350-bib-0035] Nett, M. , H. Ikeda , and B. S. Moore . 2009. “Genomic Basis for Natural Product Biosynthetic Diversity in the Actinomycetes.” Natural Product Reports 26: 1362–1384. 10.1039/B817069J. 19844637 PMC3063060

[ece374350-bib-0036] Nguyen, L. T. , H. A. Schmidt , A. Von Haeseler , and B. Q. Minh . 2015. “IQ‐TREE: A Fast and Effective Stochastic Algorithm for Estimating Maximum‐Likelihood Phylogenies.” Molecular Biology and Evolution 32: 268–274. 10.1093/molbev/msu300. 25371430 PMC4271533

[ece374350-bib-0037] Ōmura, S. , H. Ikeda , J. Ishikawa , et al. 2001. “Genome Sequence of an Industrial Microorganism *Streptomyces avermitilis* : Deducing the Ability of Producing Secondary Metabolites.” Proceedings of the National Academy of Sciences 98: 12215–12220. 10.1073/pnas.211433198. PMC59794 11572948

[ece374350-bib-0038] Ondov, B. D. , N. H. Bergman , and A. M. Phillippy . 2015. “Krona: Interactive Metagenomic Visualization in a Web Browser.” In Encyclopedia of Metagenomics, 339–346. Springer. 10.1186/1471-2105-12-385 PMC3190407 21961884

[ece374350-bib-0039] Page, A. J. , C. A. Cummins , M. Hunt , et al. 2015. “Roary: Rapid Large‐Scale Prokaryote Pan Genome Analysis.” Bioinformatics 31: 3691–3693. 10.1093/bioinformatics/btv421. 26198102 PMC4817141

[ece374350-bib-0040] Passari, A. K. , C. Caicedo‐Montoya , M. Manzo‐Ruiz , et al. 2025. “Comparative Genomics of the Streptomyces Genus: Insights Into Multi‐Stress‐Resistant Genes for Bioremediation.” World Journal of Microbiology and Biotechnology 41: 492. 10.1007/s11274-025-04703-1. 41329251 PMC12672707

[ece374350-bib-0041] Qi, Y. , K. K. Nepal , and J. A. V. Blodgett . 2021. “A Comparative Metabologenomic Approach Reveals Mechanistic Insights Into Streptomyces Antibiotic Crypticity.” Proceedings of the National Academy of Sciences of the United States of America 118: e2103515118. 10.1073/pnas.2103515118. 34326261 PMC8346890

[ece374350-bib-0042] Rasko, D. A. , M. J. Rosovitz , G. S. Myers , et al. 2008. “The Pangenome Structure of *Escherichia coli* : Comparative Genomic Analysis of *E. coli* Commensal and Pathogenic Isolates.” Journal of Bacteriology 190: 6881–6893. 10.1128/jb.00619-08. 18676672 PMC2566221

[ece374350-bib-0043] Rosenberg, E. , E. F. DeLong , S. Lory , E. Stackebrandt , and F. Thompson . 2014. The Prokaryotes: Deltaproteobacteria and Epsilonproteobacteria. Springer.

[ece374350-bib-0044] Savi, D. C. , C. W. I. Haminiuk , G. T. S. Sora , et al. 2015. “Antitumor, Antioxidant and Antibacterial Activities of Secondary Metabolites Extracted by Endophytic Actinomycetes Isolated From Vochysia Divergens.” International Journal of Pharmaceutical Chemistry and Biological Sciences 5, no. 1: 347–356.

[ece374350-bib-0045] Seemann, T. 2014. “Prokka: Rapid Prokaryotic Genome Annotation.” Bioinformatics 30: 2068–2069. 10.1093/bioinformatics/btu153. 24642063

[ece374350-bib-0046] Shannon, P. , A. Markiel , O. Ozier , et al. 2003. “Cytoscape: A Software Environment for Integrated Models of Biomolecular Interaction Networks.” Genome Research 13: 2498–2504. 10.1101/gr.1239303. 14597658 PMC403769

[ece374350-bib-0047] Shen, W. , S. Le , Y. Li , and F. Hu . 2016. “SeqKit: A Cross‐Platform and Ultrafast Toolkit for FASTA/Q File Manipulation.” PLoS One 11: e0163962. 10.1371/journal.pone.0163962. 27706213 PMC5051824

[ece374350-bib-0048] Shen, W. , and H. Ren . 2021. “TaxonKit: A Practical and Efficient NCBI Taxonomy Toolkit.” Journal of Genetics and Genomics 48: 844–850. 10.1016/j.jgg.2021.03.006. 34001434

[ece374350-bib-0049] Stackebrandt, E. , F. A. Rainry , and N. L. Ward‐Rainey . 1997. “Proposal for a New Hierarchic Classification System, Actinobacteria Classis nov.” International Journal of Systematic and Evolutionary Microbiology 47: 479–491. 10.1099/00207713-47-2-479.

[ece374350-bib-0050] Tang, H. , V. Krishnakumar , X. Zeng , et al. 2024. “JCVI: A Versatile Toolkit for Comparative Genomics Analysis.” iMeta 3: e211. 10.1002/imt2.211. 39135687 PMC11316928

[ece374350-bib-0051] Tegenfeldt, F. , D. Kuznetsov , M. Manni , M. Berkeley , E. M. Zdobnov , and E. V. Kriventseva . 2025. “OrthoDB and BUSCO Update: Annotation of Orthologs With Wider Sampling of Genomes.” Nucleic Acids Research 53: D516–D522. 10.1093/nar/gkae987. 39535043 PMC11701741

[ece374350-bib-0052] Tettelin, H. , V. Masignani , M. J. Cieslewicz , et al. 2005. “Genome Analysis of Multiple Pathogenic Isolates of *Streptococcus agalactiae* : Implications for the Microbial “Pan‐Genome”.” Proceedings of the National Academy of Sciences of the United States of America 102: 13950–13955. 10.1073/pnas.0506758102. 16172379 PMC1216834

[ece374350-bib-0053] Wang, D. , Y. Zhang , Z. Zhang , J. Zhu , and J. Yu . 2010. “KaKs_Calculator 2.0: A Toolkit Incorporating Gamma‐Series Methods and Sliding Window Strategies.” Genomics, Proteomics & Bioinformatics 8: 77–80. 10.1016/S1672-0229(10)60008-3. PMC5054116 20451164

[ece374350-bib-0054] Westhoff, S. , A. M. Kloosterman , S. F. A. van Hoesel , G. P. van Wezel , and D. E. Rozen . 2021. “Competition Sensing Changes Antibiotic Production in *Streptomyces* .” MBio 12: e02729‐20. 10.1128/mbio.02729-20. PMC7885098 33563841

[ece374350-bib-0055] Wick, R. R. , L. M. Judd , C. L. Gorrie , and K. E. Holt . 2017. “Unicycler: Resolving Bacterial Genome Assemblies From Short and Long Sequencing Reads.” PLoS Computational Biology 13: e1005595. 10.1371/journal.pcbi.1005595. 28594827 PMC5481147

[ece374350-bib-0056] Wright, F. , and M. J. Bibb . 1992. “Codon Usage in the G+C‐Rich Streptomyces Genome.” Gene 113: 55–65. 10.1016/0378-1119(92)90669-G. 1563633

[ece374350-bib-0057] Xie, C. , X. Mao , J. Huang , et al. 2011. “KOBAS 2.0: A Web Server for Annotation and Identification of Enriched Pathways and Diseases.” Nucleic Acids Research 39: W316–W322. 10.1093/nar/gkr483. 21715386 PMC3125809

[ece374350-bib-0058] Yoon‐Hee, C. , K. Hiyoung , J. Chang‐Hun , et al. 2021. “Comparative Genomics Reveals a Remarkable Biosynthetic Potential of the Streptomyces Phylogenetic Lineage Associated With Rugose‐Ornamented Spores.” mSystems 6: e0048921. 10.1128/msystems.00489-21. 34427515 PMC8407293

[ece374350-bib-0059] Yu, G. , L.‐G. Wang , Y. Han , and Q.‐Y. He . 2012. “clusterProfiler: An R Package for Comparing Biological Themes Among Gene Clusters.” OMICS: A Journal of Integrative Biology 16: 284–287. 10.1089/omi.2011.0118. 22455463 PMC3339379

[ece374350-bib-0060] Zeng, L. , D. Wang , N. Hu , et al. 2017. “A Novel Pan‐Genome Reverse Vaccinology Approach Employing a Negative‐Selection Strategy for Screening Surface‐Exposed Antigens Against *Leptospirosi*s.” Frontiers in Microbiology 8: 396. 10.3389/fmicb.2017.00396. 28352257 PMC5348505

[ece374350-bib-0061] Zhao, M. , W. Zhang , C. Yang , et al. 2024. “Discovery of Kebanmycins With Antibacterial and Cytotoxic Activities From the Mangrove‐Derived Streptomyces sp. SCSIO 40068.” Journal of Natural Products 87: 1591–1600. 10.1021/acs.jnatprod.4c00232. 38862138

[ece374350-bib-0062] Zhou, D. , T. Jing , Y. Chen , et al. 2022. “Biocontrol Potential of a Newly Isolated Streptomyces sp. HSL‐9B From Mangrove Forest on Postharvest Anthracnose of Mango Fruit Caused by Colletotrichum Gloeosporioides.” Food Control 135: 108836. 10.1016/j.foodcont.2022.108836.

[ece374350-bib-0063] Zhou, X. 2022. “Mangrove Soil‐Derived Streptomyces: An Important Resource of Pharmaceutical Active Natural Products.” Journal of Holistic Integrative Pharmacy 3: 300–314. 10.1016/S2707-3688(23)00050-X.

[ece374350-bib-0064] Zhu, Q. , M. Kosoy , and K. Dittmar . 2014. “HGTector: An Automated Method Facilitating Genome‐Wide Discovery of Putative Horizontal Gene Transfers.” BMC Genomics 15: 717. 10.1186/1471-2164-15-717. 25159222 PMC4155097

