# Supplementary material for: Pangenome of Streptomyces sampsonii and Relatives Highlights Horizontal Gene Transfer and Secondary Metabolism in Environmental Adaptation and Ecological Significance
Source: Ecol Evol. 2026 Sep 15;16(9):e74350. doi: 10.1002/ece3.74350 (PMC13577786; doi:10.1002/ece3.74350)
Supplement: Supplementary file 1 [file ECE3-16-e74350-s003.pdf]

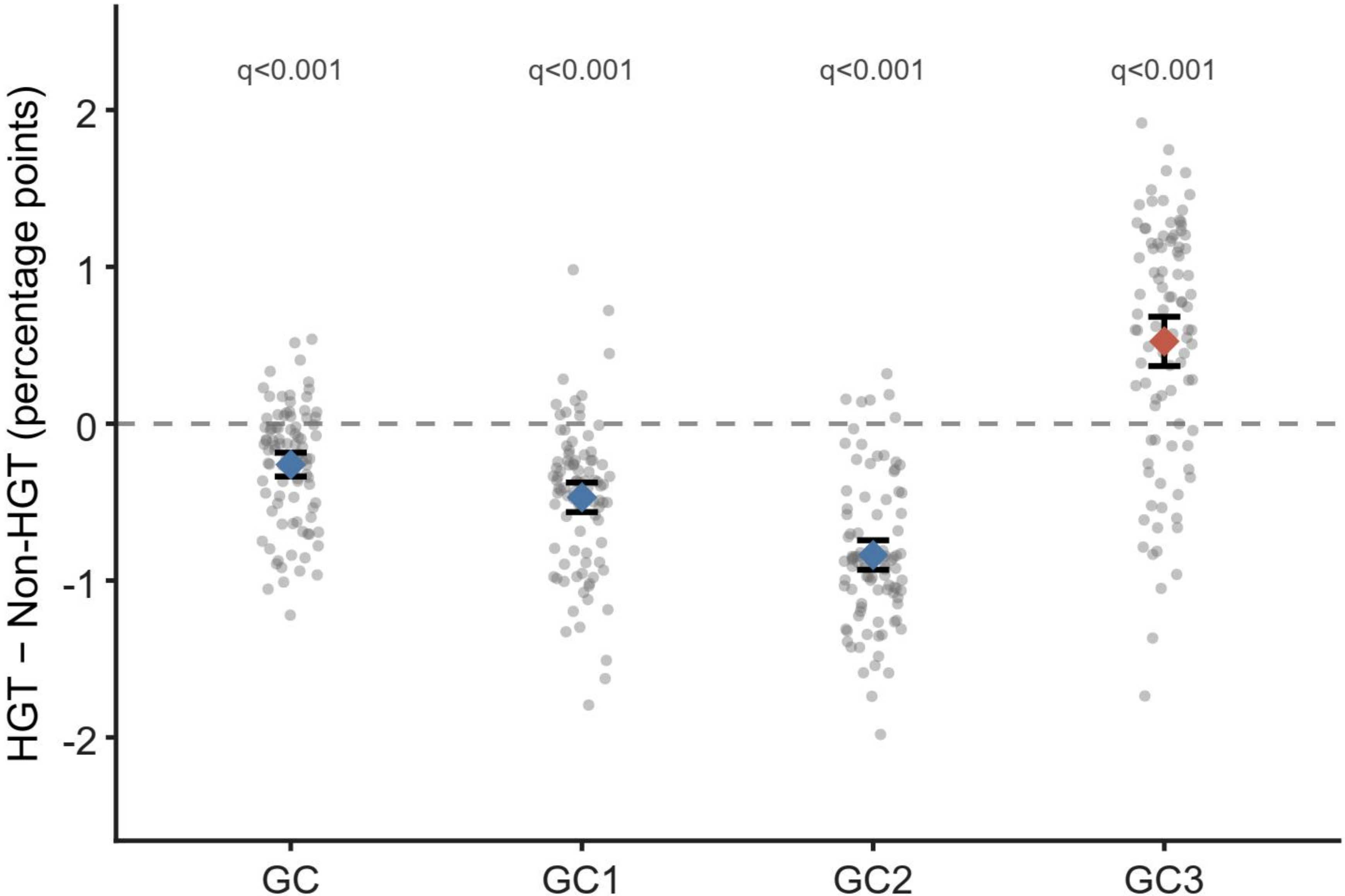
q<0.001
q<0.001
q<0.001
q<0.001
HGT – Non-HGT (percentage points)
2
1
0
-1
-2
GC
GC1
GC2
GC3
