# Supplementary material for: Pangenome of Streptomyces sampsonii and Relatives Highlights Horizontal Gene Transfer and Secondary Metabolism in Environmental Adaptation and Ecological Significance
Source: Ecol Evol. 2026 Sep 15;16(9):e74350. doi: 10.1002/ece3.74350 (PMC13577786; doi:10.1002/ece3.74350)
Supplement: Supplementary file 2 [file ECE3-16-e74350-s004.pdf]

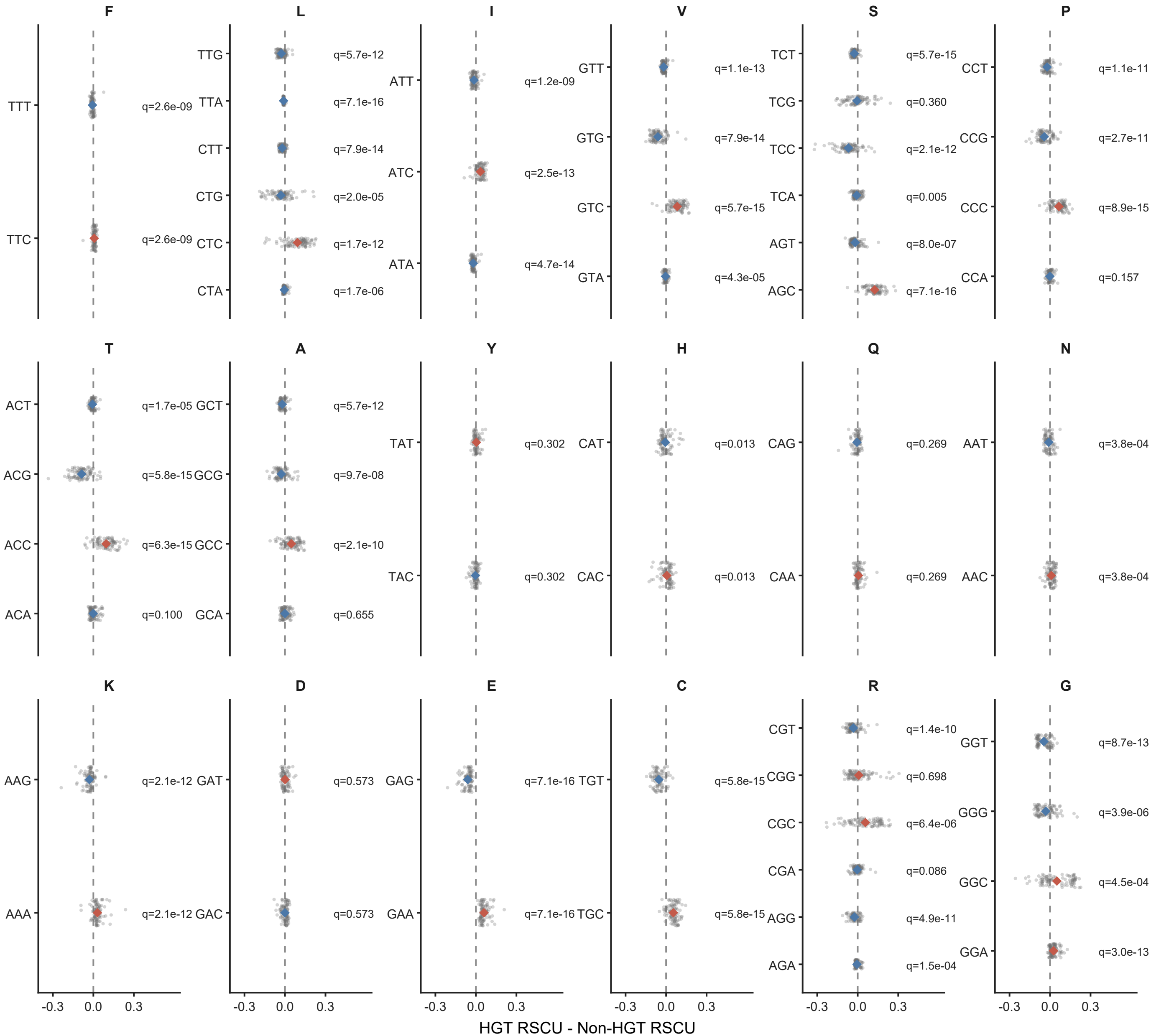

F
TTT q=2.6e-09
TTC q=2.6e-09
L
TTG q=5.7e-12
TTA q=7.1e-16
CTT q=7.9e-14
CTG q=2.0e-05
CTC q=1.7e-12
CTA q=1.7e-06
I
ATT q=1.2e-09
ATC q=2.5e-13
ATA q=4.7e-14
V
GTT q=1.1e-13
GTG q=7.9e-14
GTC q=5.7e-15
GTA q=4.3e-05
S
TCT q=5.7e-15
TCG q=0.360
TCC q=2.1e-12
TCA q=0.005
AGT q=8.0e-07
AGC q=7.1e-16
P
CCT q=1.1e-11
CCG q=2.7e-11
CCC q=8.9e-15
CCA q=0.157
T
ACT q=1.7e-05
ACG q=5.8e-15
ACC q=6.3e-15
ACA q=0.100
A
GCT q=5.7e-12
GCG q=9.7e-08
GCC q=2.1e-10
GCA q=0.655
Y
TAT q=0.302
TAC q=0.302
H
CAT q=0.013
CAC q=0.013
Q
CAG q=0.269
CAA q=0.269
N
AAT q=3.8e-04
AAC q=3.8e-04
K
AAG q=2.1e-12
AAA q=2.1e-12
D
GAT q=0.573
GAC q=0.573
E
GAG q=7.1e-16
GAA q=7.1e-16
C
TGT q=5.8e-15
TGC q=5.8e-15
R
CGT q=1.4e-10
CGG q=0.698
CGC q=6.4e-06
CGA q=0.086
AGG q=4.9e-11
AGA q=1.5e-04
G
GGT q=8.7e-13
GGG q=3.9e-06
GGC q=4.5e-04
GGA q=3.0e-13
-0.3 0.0 0.3
HGT RSCU - Non-HGT RSCU
