# Supplementary material for: Pangenome of Streptomyces sampsonii and Relatives Highlights Horizontal Gene Transfer and Secondary Metabolism in Environmental Adaptation and Ecological Significance
Source: Ecol Evol. 2026 Sep 15;16(9):e74350. doi: 10.1002/ece3.74350 (PMC13577786; doi:10.1002/ece3.74350)
Supplement: Supplementary file 3 [file ECE3-16-e74350-s007.pdf]

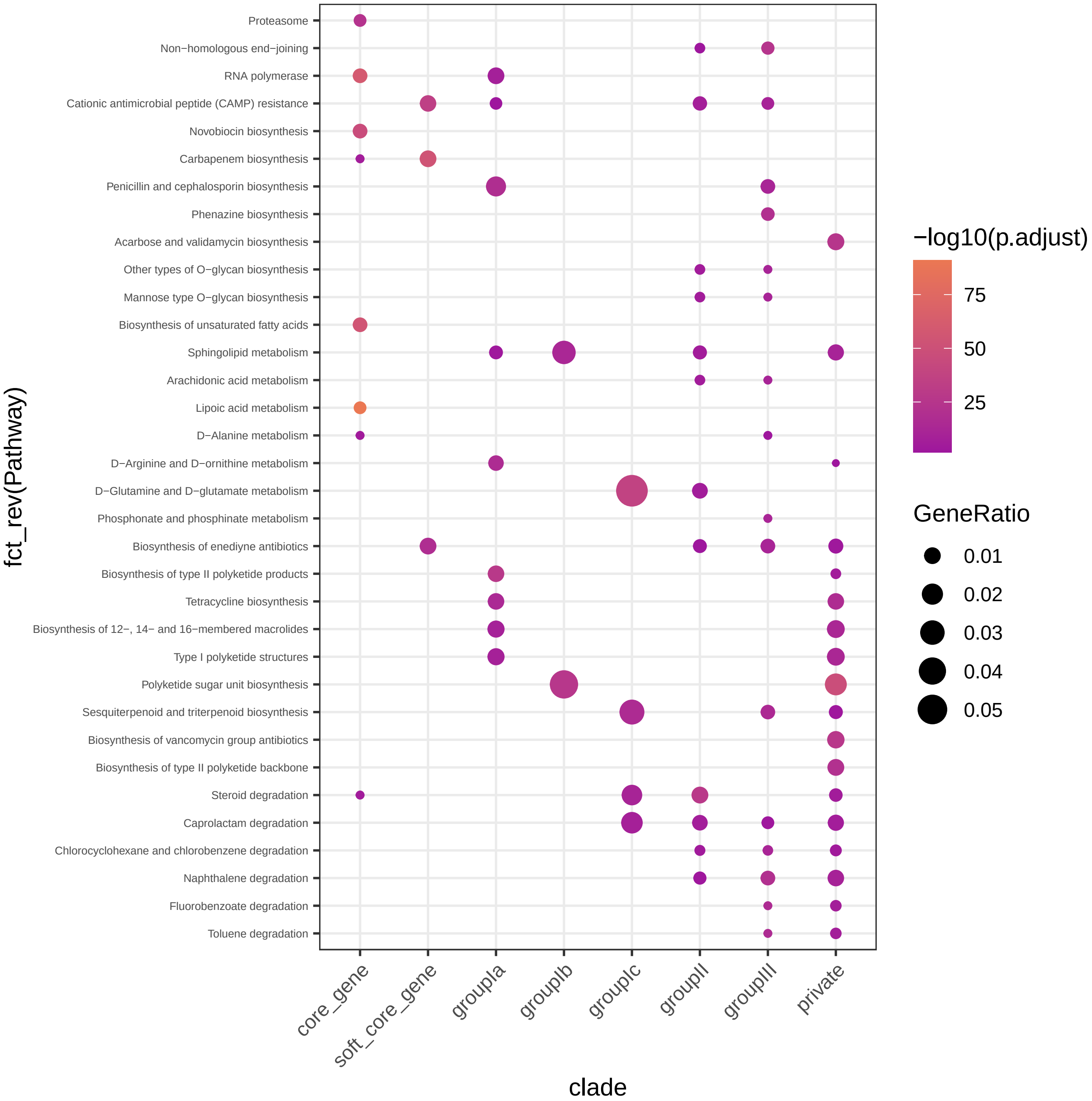
Proteasome
Non−homologous end−joining
RNA polymerase
Cationic antimicrobial peptide (CAMP) resistance
Novobiocin biosynthesis
Carbapenem biosynthesis
Penicillin and cephalosporin biosynthesis
Phenazine biosynthesis
Acarbose and validamycin biosynthesis
Other types of O−glycan biosynthesis
Mannose type O−glycan biosynthesis
Biosynthesis of unsaturated fatty acids
Sphingolipid metabolism
Arachidonic acid metabolism
Lipoic acid metabolism
D−Alanine metabolism
D−Arginine and D−ornithine metabolism
D−Glutamine and D−glutamate metabolism
Phosphonate and phosphinate metabolism
Biosynthesis of enediyne antibiotics
Biosynthesis of type II polyketide products
Tetracycline biosynthesis
Biosynthesis of 12−, 14− and 16−membered macrolides
Type I polyketide structures
Polyketide sugar unit biosynthesis
Sesquiterpenoid and triterpenoid biosynthesis
Biosynthesis of vancomycin group antibiotics
Biosynthesis of type II polyketide backbone
Steroid degradation
Caprolactam degradation
Chlorocyclohexane and chlorobenzene degradation
Naphthalene degradation
Fluorobenzoate degradation
Toluene degradation
fct_rev(Pathway)
core_gene
soft_core_gene
groupIa
groupIb
groupIc
groupII
groupIII
private
clade
−log10(p.adjust)
75
50
25
GeneRatio
0.01
0.02
0.03
0.04
0.05
