# Supplementary material for: Pangenome of Streptomyces sampsonii and Relatives Highlights Horizontal Gene Transfer and Secondary Metabolism in Environmental Adaptation and Ecological Significance
Source: Ecol Evol. 2026 Sep 15;16(9):e74350. doi: 10.1002/ece3.74350 (PMC13577786; doi:10.1002/ece3.74350)
Supplement: Supplementary file 4 [file ECE3-16-e74350-s009.pdf]

Strain	Clade	KJ4	MCCC 1A01650
Streptomyces rochei JK1	Clade I	71.4	71.4
Streptomyces rochei SR1102	Clade I	71.4	71.4
Streptomyces enissocaesilis NBF 715	Clade I	71.4	71.5
Streptomyces rochei D21E05	Clade I	71.4	71.4
Streptomyces mutabilis MPB24	Clade I	71.4	71.4
Streptomyces rochei S32	Clade I	71.3	71.3
Streptomyces albogriseolus LBX-2	Clade I	70.9	70.9
Streptomyces rubrogriseus NBRC 15455	Clade I	70.9	70.9
Streptomyces lienomycini DSM 41475	Clade I	70.7	70.7
Streptomyces viridosporus ATCC 39115	Clade I	70.9	70.9
Streptomyces anthocyanicus NBC 01687	Clade I	70.7	70.7
Streptomyces tuirus JCM 4255	Clade I	71.1	71.1
Streptomyces anthocyanicus NBC 01777	Clade I	70.7	70.7
Streptomyces anthocyanicus NBC 00116	Clade I	70.7	70.7
Streptomyces tendae 139	Clade I	70.7	70.7
Streptomyces anthocyanicus NBC 01636	Clade I	70.7	70.7
Streptomyces anthocyanicus NBC 01662	Clade I	70.7	70.7
Streptomyces violaceoruber CGMCC 4.1801	Clade I	70.7	70.7
Streptomyces anthocyanicus NBC 01372	Clade I	70.7	70.7
Streptomyces nigra LM01	Clade I	70.8	70.8
Streptomyces olivaceus SCSIO T05	Clade I	70.8	70.8
Streptomyces collinus L2	Clade I	70.6	70.7
Streptomyces nigra 452	Clade I	70.8	70.8
Streptomyces collinus SQ	Clade I	70.6	70.7
Streptomyces olivaceus B-81	Clade I	70.8	70.8
Streptomyces nigra NBC 00206	Clade I	70.8	70.8
Streptomyces olivaceus KLBMP 5084	Clade I	70.6	70.6
Streptomyces collinus NA01503	Clade I	70.9	70.9
Streptomyces griseoincarnatus HNS054	Clade I	70.4	70.4
Streptomyces iakyrus CGMCC 4.1912	Clade I	71.0	71.0
Streptomyces collinus Tu 365 Tu 365	Clade I	70.6	70.7
Streptomyces collinus Inha504	Clade I	70.6	70.7
Streptomyces collinus CGMCC 4.1623	Clade I	70.8	70.8
Streptomyces albogriseolus A36	Clade I	69.9	69.9
Streptomyces nigra MT6	Clade I	70.1	70.1
Streptomyces okerensis DSM 116494	Clade I	70.3	70.3
Streptomyces cadmiisoli ZFG47	Clade I	70.7	70.8
Streptomyces rochei 7434AN4	Clade I	70.4	70.3
Streptomyces lavenduligriseus Tu901	Clade I	70.2	70.2
Streptomyces violaceoruber BM-41	Clade II	69.3	69.5
Streptomyces violaceoruber S21	Clade II	69.4	69.5
Streptomyces microflavus NBC 01391	Clade II	69.3	69.5
Streptomyces microflavus NBC 01595	Clade II	69.3	69.5
Streptomyces microflavus NBC 01346	Clade II	69.3	69.4
Streptomyces microflavus DSM 40593	Clade II	69.2	69.3
Streptomyces microflavus NBC 01683	Clade II	69.3	69.3
Streptomyces microflavus NA06532	Clade II	69.4	69.5
Streptomyces microflavus NBC 01798	Clade II	69.3	69.3
Streptomyces microflavus DF	Clade II	69.2	69.4
Streptomyces microflavus AM6	Clade II	69.3	69.3
Streptomyces microflavus AM4	Clade II	69.2	69.2
Streptomyces microflavus NBC 01684	Clade II	69.0	69.1
Streptomyces microflavus NBC 01184	Clade II	69.0	69.1
Streptomyces microflavus NBC 01194	Clade II	69.1	69.2
Streptomyces microflavus NBC 01168	Clade II	69.0	69.1
Streptomyces albidoflavus W68	Clade III	95.0	95.0
Streptomyces albidoflavus UYFA156	Clade III	95.0	95.0
Streptomyces albidoflavus NBC 01671	Clade III	95.0	95.0
Streptomyces albidoflavus MGMM6	Clade III	95.0	95.0
Streptomyces albidoflavus NBC 01170	Clade III	95.0	95.0
Streptomyces albidoflavus NBC 01665	Clade III	95.0	95.0
Streptomyces albidoflavus NBC 01722	Clade III	95.0	95.0
Streptomyces albidoflavus RKJM-0023	Clade III	95.0	95.0
Streptomyces albidoflavus NBC 01675	Clade III	95.0	95.0
Streptomyces albidoflavus NBC 01110	Clade III	95.0	95.0
Streptomyces albidoflavus NBC 01673	Clade III	95.0	95.0
Streptomyces albidoflavus LGO-A23	Clade III	95.0	95.0
Streptomyces albidoflavus NBC 01271	Clade III	95.0	95.0
Streptomyces albidoflavus J1074 D14	Clade III	95.0	95.0
Streptomyces albidoflavus J1074 D14 4N24	Clade III	95.0	95.0
Streptomyces albidoflavus NBC 01791	Clade III	95.0	95.0
Streptomyces albidoflavus LGO-A16	Clade III	95.0	95.0
Streptomyces albidoflavus NBC 01640	Clade III	95.0	95.0
Streptomyces albidoflavus SM254	Clade III	95.0	95.0
Streptomyces albidoflavus J1074 R2	Clade III	95.0	95.0
Streptomyces albidoflavus NBC 01627	Clade III	95.0	95.0
Streptomyces albidoflavus J1074	Clade III	95.0	95.0
Streptomyces albidoflavus NBC 01270	Clade III	95.0	95.0
Streptomyces albidoflavus NBC 01621	Clade III	95.0	95.0
Streptomyces albidoflavus NBC 01374	Clade III	95.0	95.0
Streptomyces albidoflavus NBC 01616	Clade III	95.0	95.0
Streptomyces albidoflavus NBC 01747	Clade III	95.0	95.0
Streptomyces albidoflavus NBC 01692	Clade III	95.0	95.0
Streptomyces albidoflavus ATCC 23899	Clade III	95.0	95.0
Streptomyces albidoflavus NBC 01664	Clade III	95.0	95.0
Streptomyces albidoflavus NBC 01661	Clade III	95.0	95.0
Streptomyces albidoflavus NBC 01274	Clade III	95.0	95.0
Streptomyces albidoflavus CCOS 2040	Clade III	95.0	95.0
Streptomyces albidoflavus NBC 01331	Clade III	95.0	95.0
Streptomyces albidoflavus SC-3	Clade III	95.0	95.0
Streptomyces albidoflavus S20	Clade III	95.0	95.0
Streptomyces albidoflavus NBC 01790	Clade III	95.0	95.0
Streptomyces albidoflavus NBC 01658	Clade III	95.0	95.0

AAI (%): 70 – 75 – 80 – 85 – 90 – 95

Clade: Clade I, Clade II, Clade III

Genome Group	Clade	KJ40	MCCC 1A01650
Streptomyces rochei JK1	Clade I	82.1	82.1
Streptomyces rochei SR1102	Clade I	82.1	82.1
Streptomyces enissocaesilis NBF 715	Clade I	82.0	82.0
Streptomyces rochei D21E05	Clade I	82.0	82.1
Streptomyces mutabilis MPB24	Clade I	82.0	82.1
Streptomyces rochei S32	Clade I	82.0	82.1
Streptomyces albogriseolus LBX-2	Clade I	81.9	81.9
Streptomyces rubrogriseus NBRC 15455	Clade I	81.8	81.9
Streptomyces lienomycini DSM 41475	Clade I	81.9	82.0
Streptomyces viridosporus ATCC 39115	Clade I	81.9	81.8
Streptomyces anthocyanicus NBC 01687	Clade I	81.9	81.8
Streptomyces tuirus JCM 4255	Clade I	81.6	81.6
Streptomyces anthocyanicus NBC 01777	Clade I	81.9	81.8
Streptomyces anthocyanicus NBC 00116	Clade I	81.9	81.8
Streptomyces tendae 139	Clade I	81.9	81.8
Streptomyces anthocyanicus NBC 01636	Clade I	81.9	81.8
Streptomyces anthocyanicus NBC 01662	Clade I	81.9	81.8
Streptomyces violaceoruber CGMCC 4.1801	Clade I	81.8	81.8
Streptomyces anthocyanicus NBC 01372	Clade I	81.8	81.8
Streptomyces nigra LM01	Clade I	81.7	81.7
Streptomyces olivaceus SCSIO T05	Clade I	81.7	81.7
Streptomyces collinus L2	Clade I	81.8	81.8
Streptomyces nigra 452	Clade I	81.7	81.7
Streptomyces collinus SQ	Clade I	81.9	81.7
Streptomyces olivaceus B-81	Clade I	81.7	81.6
Streptomyces nigra NBC 00206	Clade I	81.7	81.6
Streptomyces olivaceus KLBMP 5084	Clade I	81.8	81.7
Streptomyces collinus NA01503	Clade I	81.4	81.6
Streptomyces griseoincarnatus HNS054	Clade I	81.7	81.8
Streptomyces iakyrus CGMCC 4.1912	Clade I	81.3	81.5
Streptomyces collinus Tu 365 Tu 365	Clade I	81.6	81.5
Streptomyces collinus Inha504	Clade I	81.6	81.4
Streptomyces collinus CGMCC 4.1623	Clade I	81.3	81.5
Streptomyces albogriseolus A36	Clade I	81.8	81.8
Streptomyces nigra MT6	Clade I	81.6	81.7
Streptomyces okerensis DSM 116494	Clade I	81.5	81.4
Streptomyces cadmiisoli ZFG47	Clade I	81.2	81.1
Streptomyces rochei 7434AN4	Clade I	81.4	81.4
Streptomyces lavenduligriseus Tu901	Clade I	81.4	81.3
Streptomyces violaceoruber BM-41	Clade II	82.0	82.0
Streptomyces violaceoruber S21	Clade II	82.0	81.9
Streptomyces microflavus NBC 01391	Clade II	82.0	81.9
Streptomyces microflavus NBC 01595	Clade II	82.0	81.9
Streptomyces microflavus NBC 01346	Clade II	81.9	81.9
Streptomyces microflavus DSM 40593	Clade II	82.0	81.9
Streptomyces microflavus NBC 01683	Clade II	82.0	81.8
Streptomyces microflavus NA06532	Clade II	81.9	81.8
Streptomyces microflavus NBC 01798	Clade II	81.9	81.9
Streptomyces microflavus DF	Clade II	81.9	81.9
Streptomyces microflavus AM6	Clade II	81.9	81.9
Streptomyces microflavus AM4	Clade II	81.9	81.9
Streptomyces microflavus NBC 01684	Clade II	82.0	81.9
Streptomyces microflavus NBC 01184	Clade II	82.0	81.8
Streptomyces microflavus NBC 01194	Clade II	81.9	81.8
Streptomyces microflavus NBC 01168	Clade II	81.9	81.8
Streptomyces albidoflavus W68	Clade III	96.2	99.5
Streptomyces albidoflavus UYFA156	Clade III	96.2	99.4
Streptomyces albidoflavus NBC 01671	Clade III	96.1	99.5
Streptomyces albidoflavus MGMM6	Clade III	98.9	96.3
Streptomyces albidoflavus NBC 01170	Clade III	98.8	96.3
Streptomyces albidoflavus NBC 01665	Clade III	98.8	96.2
Streptomyces albidoflavus NBC 01722	Clade III	98.8	96.3
Streptomyces albidoflavus RKJM-0023	Clade III	98.8	96.2
Streptomyces albidoflavus NBC 01675	Clade III	98.8	96.3
Streptomyces albidoflavus NBC 01110	Clade III	98.8	96.3
Streptomyces albidoflavus NBC 01673	Clade III	98.8	96.2
Streptomyces albidoflavus LGO-A23	Clade III	98.8	96.2
Streptomyces albidoflavus NBC 01271	Clade III	98.8	96.3
Streptomyces albidoflavus J1074 D14	Clade III	98.8	96.2
Streptomyces albidoflavus J1074 D14 4N24	Clade III	98.8	96.2
Streptomyces albidoflavus NBC 01791	Clade III	98.8	96.2
Streptomyces albidoflavus LGO-A16	Clade III	98.8	96.2
Streptomyces albidoflavus NBC 01640	Clade III	98.8	96.2
Streptomyces albidoflavus SM254	Clade III	98.8	96.2
Streptomyces albidoflavus J1074 R2	Clade III	98.8	96.2
Streptomyces albidoflavus NBC 01627	Clade III	98.8	96.2
Streptomyces albidoflavus J1074	Clade III	98.8	96.2
Streptomyces albidoflavus NBC 01270	Clade III	98.8	96.2
Streptomyces albidoflavus NBC 01621	Clade III	98.8	96.2
Streptomyces albidoflavus NBC 01374	Clade III	98.8	96.2
Streptomyces albidoflavus NBC 01616	Clade III	98.8	96.2
Streptomyces albidoflavus NBC 01747	Clade III	98.7	96.2
Streptomyces albidoflavus NBC 01692	Clade III	98.8	96.2
Streptomyces albidoflavus ATCC 23899	Clade III	98.8	96.2
Streptomyces albidoflavus NBC 01664	Clade III	98.8	96.2
Streptomyces albidoflavus NBC 01661	Clade III	98.8	96.2
Streptomyces albidoflavus NBC 01274	Clade III	98.8	96.2
Streptomyces albidoflavus CCOS 2040	Clade III	98.8	96.2
Streptomyces albidoflavus NBC 01331	Clade III	98.7	96.1
Streptomyces albidoflavus SC-3	Clade III	98.7	96.2
Streptomyces albidoflavus S20	Clade III	96.0	96.0
Streptomyces albidoflavus NBC 01790	Clade III	96.0	95.9
Streptomyces albidoflavus NBC 01658	Clade III	95.7	95.7

ANI (%): 85 – 90 – 95 – 100

Clade: Clade I, Clade II, Clade III
